# Potentials of synthetic hexaploid wheats to improve drought tolerance

**DOI:** 10.1038/s41598-022-24678-5

**Published:** 2022-11-28

**Authors:** Niloofar Mokhtari, Mohammad Mahdi Majidi, Aghafakhr Mirlohi

**Affiliations:** Department of Agronomy and Plant Breeding, College of Agriculture, Isfahan University of Technology, Isfahan, 8415683111 Iran

**Keywords:** Plant breeding, Plant domestication, Plant stress responses

## Abstract

Synthetic hexaploid wheat-derived lines (SHW-DL) offers new hope for breeders to restore genes lost during the evolutionary bottleneck. The study of adaptability, variation, and the possibility of selection in SHW-DL for drought tolerance is poorly understood in arid environments. The potential of 184 SHW-DL and their variation for agro-morphological traits were assessed under normal and water stress conditions for 2 years. The mean values of grain yield (YLD) varied from 683.9 g/m^2^ (water stress) to 992.1 g/m^2^ (normal conditions). Grain yield decreased by 64 and 71% under water stress in the two growing seasons. High genotypic variation was found for measured traits and drought tolerance. Heritability ranged from 19 (harvest index) to 47% (spike length), whereas grain yield indicated a moderate heritability (32%). Using the assessment of the interrelationship of traits, hectoliter (a quality trait) was correlated with drought tolerance and stability indices. Therefore, it can be considered as an important trait to select drought tolerant genotypes. In the following, the priority of yield components entering the regression model was different in two moisture conditions suggesting different strategies in indirect selection programs to improve yield. Spike m^−2^ and grain spike^−1^ indirectly and negatively affected yield through thousand-grain weight (TGW) under normal and water stress conditions, respectively. Furthermore, SHW-DL compared to ordinary wheat were significantly superior in terms of early maturity, dwarfing, yield, TGW, stem diameter, and harvest index. Overall, our findings suggest that SHW-DL are a valuable source for improving wheat yield and drought tolerance, and indirect selection might be possible to improve these complex traits.

## Introduction

**Figure 1:**
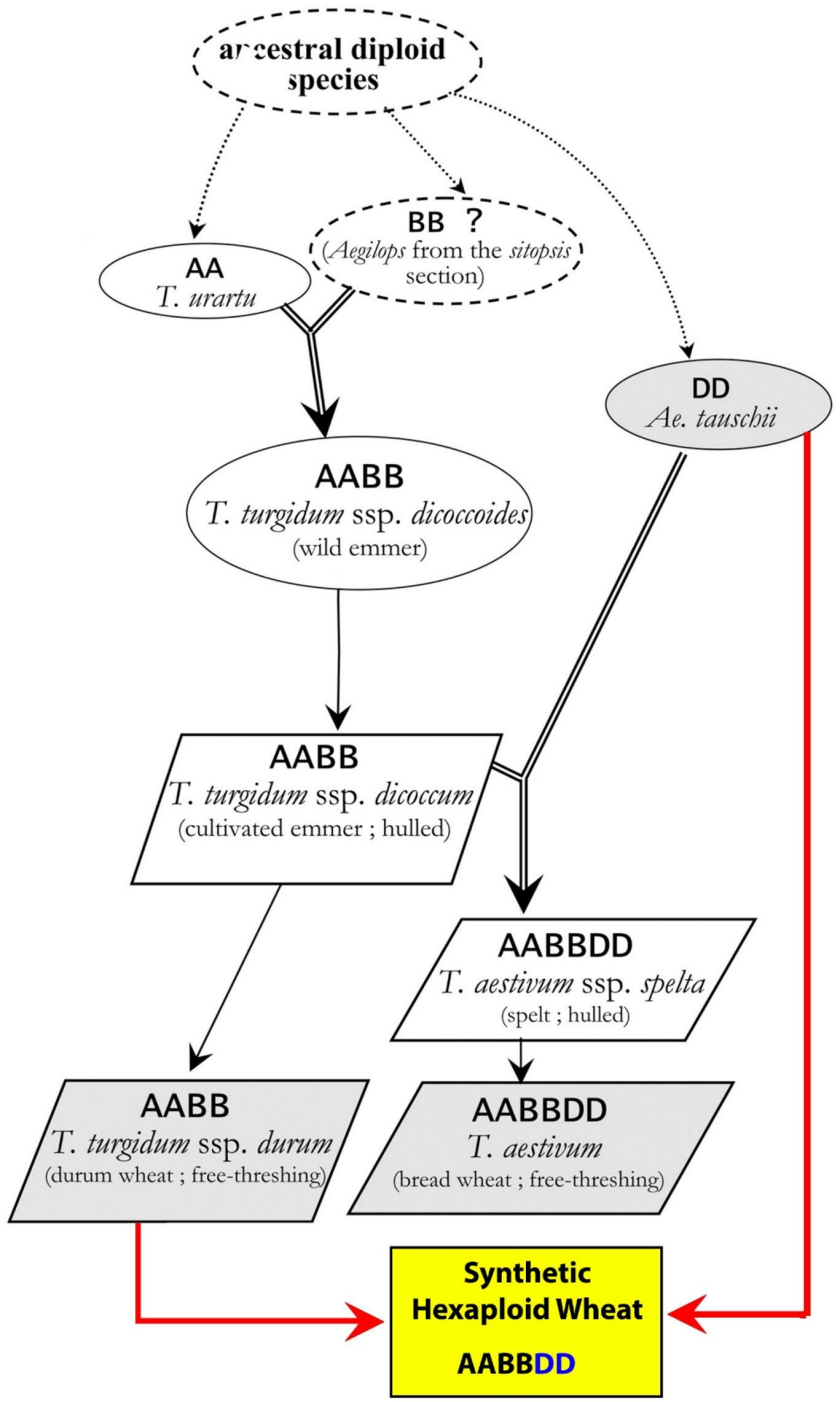
Development of synthetic hexaploid wheat (AABBDD) in comparison with the emulating evolution of the hexaploid wheat (AABBDD).

Bread wheat (*Triticum aestivum* L.) is one of the most important cereal crops that evolved from the hybridization of *T. turgidum* (AABB) with *Aegilops tauschii* (DD)^1, 2^. Common wheat has gone through a genetic bottleneck experienced during domestication and evolved with a narrow genetic variation. Therefore, compared to its two donor species, a considerable portion of original genetic diversity has been lost^3, 4^. Plant breeders recently suggested producing synthetic hexaploid wheat (SHW) by replicating the hybridization event that occurred between *T. turgidum* (AABB) and *Ae. tauschii* (DD) using wild types and landraces to increase the genetic diversity of modern wheat (Fig. 1)^4^.

Synthetic wheat (2n = 6x = 42, AABBDD) is artificially created using interspecific crosses between durum wheat (2n = 4x = 28, AABB, *T. turgidum* L.) and goat grass (2n = 2x = 14, DD, *Aegilops tauschii* Coss.)^1, 2^. The International Maize and Wheat Improvement Centre (CIMMYT) contains over 1000 primary SHW lines from crosses between accessions of *Ae. tauschii* (DD) and primarily modern tetraploid wheat. In addition, the primary SHW lines have been crossed to a variety of elite hexaploid wheat lines to produce synthetic-derived lines with different levels of genetic introgression from the ancestors^1^. The synthetic hexaploid wheat derived lines (SHW-DL) have been reported to have significant genetic variation for biotic and abiotic stresses resistances^5–9^, agronomic and desirable quantitative traits^10, 11^. Higher rates of photosynthesis^12^ and a significant increase in grain yield^13^ were also reported in SHW-DL. Evaluation of four SHW-DL and two spring wheat varieties under heat and drought stress conditions showed that SHW-DL might perform better in regions with repeatedly high temperatures^14^. Evaluation of 34 SHW-DL and some bread wheat also proved that SHW-DL exhibited good agronomic performance and higher activity of antioxidants under water stress^15^. Furthermore, previous research indicated that water extraction from deeper depths in drying soil increased in SHW-DL, and the productivity was higher compared to winter wheat using root morphological traits^5^. These studies suggest the great genetic potential of synthetic wheat varieties for improving yield and yield stability under drought and heat-stressed environments.

Drought is one of the most important abiotic stresses that has significantly decreased crop yield and is predicted to intensify with climate changes^16^. Drought can cause losses in different stages of wheat growth, but the most significant impact on yield reduction is during anthesis^17^. Production of drought-tolerant wheat varieties to feed the world’s growing population involves serious challenges. It is hampered by the complexity and polygenic nature of drought tolerance mechanism, lack of appropriate selection indices, and insufficient genetic diversity^18^. Selection for yield components and other yield-related traits with higher heritability may indirectly increase yield under drought stress. For example, Gororo et al.^19^ found that relatively higher-yielding cultivars in low-yielding environments had more grain filling rates and larger grains. It was also proved that grain yield, chlorophyll content, and kernel weight of synthetic wheat lines were negatively correlated with heat-susceptibility index under high-temperature conditions^8^ Therefore, introducing appropriate drought and susceptibility indices to select based on the results of both stress and non-stress conditions improves the efficiency of breeding programs to select superior genotypes^20, 21^. Several indices have been used to screen drought tolerant genotypes including SSI (Stress susceptibility index^22^), RSI (Relative stress index^23^), TOL (Tolerance^24^), MP (Mean productivity^24^), YSI (Yield stability index^25^), HM (Harmonic mean^26^), GMP (Geometric mean productivity^20^), STI (Stress tolerance index^20, 27^), YI (Yield index^28^) and finally CSI (Combination of significant indices^29^). Recently, it has been showed that the combination of the best identified tolerance and susceptibility indices are effective method (as CSI, combination of significant indices) for screening superior lines^29^.

Besides technical issues, the development of drought-tolerant wheat cultivars is also disadvantaged by the limited genetic diversity available in modern germplasm^5^. Many researchers advocate using synthetic hexaploid wheat (SHW) to mitigate the problem because it represents a broader and more comprehensive genetic base^8, 30^. Previous studies on SHW-DL have mainly focused on aspects related to the biotic stresses and less on the abiotic ones. Therefore, exploiting SHW-DL genetic potential under abiotic stresses such as drought is needed to facilitate their utilization in cultivar development, especially in arid and semiarid regions. The present study aimed to; (1) evaluate the agronomic potential and genetic diversity of 184 synthetic hexaploid wheat derived lines under different moisture environments in an arid climate of Iran, (2) identify drought tolerant lines for future studies using multivariate analysis and drought and susceptibility indices, and (3) analyze relationships between traits and possibly identify a new approach for indirect selection.

## Results

### Traits variation and heritability estimates

Results of combined ANOVA (Table S1) demonstrated significant differences (*P* < 0.01) among the evaluated lines. Moisture regimes significantly affected all the measured traits except for days to heading (DHE), plant height (PHT), awn length (AL), and spike length (SL) (*P* < 0.05). Similarly, significant differences were observed for all the features except relative water content (RWC), thousand-grain weight (TGW), and Hectoliter (He) between the two experimental years (*P* < 0.05). The results from ANOVA revealed that the effect of genotype × year was significant for all the traits excluding spike m^−2^ (SM), grains spike^−1^ (GS), He, biological yield (BY) and harvest index (HI) (*P* < 0.05) (Table S1). The mean values of genotypes decreased for all the measured traits under drought stress except DF (days to flowering) and GS (Table 1). The mean values of grain yield (YLD) varied from 683.91 g/m^2^ under water stress to 992.07 g/m^2^ under normal conditions. Grain yield decreased by 64.18% and 71.43% under water stress in the two growing seasons, respectively.

**Table 1:** Mean of agro-morphological and physiological traits evaluated under two irrigation levels (normal and stress) during 2018–2019 in synthetic hexaploid wheat derived lines.

Traits	2018		2019		Mean of 2 years	
	Normal	Stress	Normal	Stress	Normal	Stress
DHE	173.58^b^	173.97^a^	176.93^a^	176.19^b^	175.22^a^	175.10^a^
DF	185.55^b^	205.06^a^	187.64^b^	188.02^a^	186.58^b^	196.51^a^
RWC (%)	80.53^a^	56.59^b^	78.30^a^	66.85^b^	79.43^a^	61.80^b^
PHT (cm)	119.49^a^	120.47^a^	114.70^a^	110.70^b^	117.09^a^	115.46^a^
AL (cm)	5.95^a^	5.99^a^	5.67^a^	5.54^b^	5.82^a^	5.77^a^
SL (cm)	12.00^a^	12.00^a^	11.38^a^	11.31^a^	11.69^a^	11.66^a^
PL (cm)	22.96^a^	23.08^a^	20.96^a^	19.81^b^	21.98^a^	21.41^b^
SD (mm)	3.49^a^	2.90^b^	3.29^a^	2.53^b^	3.39^a^	2.72^b^
YLD (g/m^2^)	755.39^a^	484.86^b^	1229.91^a^	878.59^b^	992.07^a^	683.91^b^
SM (s/m^2^)	300.83^a^	301.82^a^	1041.76^a^	954.14^b^	671.29^a^	627.97^b^
TGW (g)	42.39^a^	27.60^b^	38.87^a^	30.32^b^	40.66^a^	29.01^b^
GS	33.16^b^	35.01^a^	39.83^b^	41.36^a^	36.49^b^	38.18^a^
He (kg/he)	82.98^a^	75.36^b^	84.41^a^	76.93^b^	82.98^a^	75.36^b^
BY (g/m^2^)	1466.13^a^	1250.90^b^	3221.87^a^	2843.02^b^	2338.88^a^	2053.56^b^
HI (%)	51.77^a^	39.56^b^	38.30^a^	31.13^b^	45.10^a^	35.33^b^

DHE, days to heading; DF, days to flowering; RWC (%), relative water content; PHT (cm), plant height; AL (cm), awn length; SL (cm), spike length; PL (cm), peduncle length; SD, stem diameter (mm); YLD (g/m^2^), grain yield; SM, spike per m^2^; TGW (g), thousand-grain weight; GS, grains per spike; He, hectoliter (kg/he); BY (g/m^2^), biological yield; HI (%), harvest index.

Means sharing no letter are significantly different at the 5% level by LSD test.

Genotypic coefficients of variation (GCV) and heritability values for all traits were calculated for both water conditions (Table 2). A high range of genetic variance was observed for biological and grain yields while a lower range was obtained for stem diameter (Table 2). The range of GCV varied from 1.39 to 20.54 for normal and 1.33 to 18.10 for water stress conditions. GCV values for DHE, DF, PHT, peduncle length (PL), SL, GS, YLD, and HI were higher in normal conditions. In contrast, DHE, DF, AL, SL, stem diameter (SD), YLD, SM, TGW, GS, and He had higher heritability under water stress conditions (Table 2). The heritability estimates ranged from 18.90 (for Harvest index) to 46.53 (for spike length) in the two water environments. DHE, DF, PHT, AL, SL PL, and SD showed high heritability but HI, BY, RWC, and SM revealed a lower value. Although the grain yield indicated a moderate heritability (32.24), the values were higher under water stress than normal conditions.

**Table 2:** Range, genotypic coefficient of variation (GCV) and broad sense heritability (h^2^) of wheat traits evaluated under normal and water-stressed conditions during 2 years.

Traits	Range (Min–Max)		GCV (%)		h^2^		Total
	Normal	Stress	Normal	Stress	Normal	Stress	
DHE	160–187	168–187	2.15	1.80	39.98	42.37	44.82
DF	174–201	182–216	1.39	1.33	39.49	41.71	44.60
RWC (%)	60.78–95	40–90	4.56	7.57	23	17.83	20.69
PHT (cm)	88–150	81.5–152	8.23	2.65	39.21	16.91	44.29
AL (cm)	1.33–8.83	1.58–8.83	17.31	18.09	42.79	44.30	46.28
SL (cm)	8.5–15.66	8.5–15.5	11.38	11.32	43.59	43.62	46.53
PL (cm)	9.66–35.53	7.6–35.8	20.54	18.10	44.41	39.03	45.56
SD (mm)	2.39–4.40	2–4.03	10	12.02	41.07	44.58	45.65
YLD (g/m^2^)	232.06–1664	202.3–1680	13.87	12.95	19.39	21.28	32.24
SM (s/m^2^)	93–1760.67	110–1962.04	13.87	14.12	19.39	21.19	28.68
TGW (g)	18.4–67.8	16.21–60.38	11.71	17.47	34.07	37.80	40.73
GS	12.58–61.66	19.98–63.16	16.05	16.02	34.55	35.49	40.48
He (kg/he)	54.49–97.5	50.21–92	3.95	6.65	29.17	35.97	39.47
BY (g/m^2^)	803–3993.04	692–3992	5.42	6.69	12.69	11.15	20.17
HI (%)	20.24–69.93	11.79–67.90	8.93	8.77	15.16	10.29	18.90

DHE, days to heading; DF, days to flowering; RWC (%), relative water content; PHT (cm), plant height; AL (cm), awn length; SL (cm), spike length; PL (cm), peduncle length; SD, stem diameter (mm); YLD, (g/m^2^) grain yield; SM, spike per m^2^; TGW (g), thousand-grain weight; GS, grains per spike; He, hectoliter (kg/he); BY (g/m^2^), biological yield; HI (%), harvest index.

### Comparison of SHW-DL with common wheat

Analysis of variance indicated significant differences between SHW-DL and common wheat lines for most studied traits (Table S1). The mean comparison related to DHE, RWC, PHT, YLD, TGW, and HI for two irrigation regimes are shown in Fig. S1. Common wheat lines showed higher days to heading, days to flowering, and plant height compared to SHW-DL in both moisture environments. Thousand-grain weight, peduncle length, stem diameter, and harvest index had higher values for SHW-DL under both moisture conditions. While SHW-DL had higher grain yield, spike m^−2^, hectoliter, and RWC in normal water environments, the difference between the two genotypic groups was not significant under water stress conditions (Fig. S1).

### Relationships of traits, indices and principal component analysis

Days to heading had a significant positive relationship with days to flowering under both normal and water stress conditions (0.86 and 0.91 for normal and stressed environments, respectively) (*P* < 0.05) (Table 3). Grain yield was positively associated with yield components (including spike m^−2^ and grain spike^−1^) under both water conditions (*P* < 0.05). Stem diameter was positively correlated with spike length and peduncle length at both water treatments. Also, there were positive correlations between grain yield with biological yield and harvest index in both water conditions. A negative correlation was observed between thousand-grain weight and grain spike^−1^ under normal water conditions. However, this relationship was positive in stress conditions (Table 3). The relationship between stress tolerance index (STI) with YLD, SM, GS, HI, and He was positive and significant under water stress conditions (*P* < 0.01). A significant positive correlation was observed between yield stability index (YSI) and YLD, SM, BY, HI, and He under water stress conditions (*P* < 0.01) (Table 3). Moreover, there was a significant positive correlation between the combination of significant indices (CSI) with YLD, SM, GS, BY, and HI (*P* < 0.01) (Table3).

**Table 3:** Correlation coefficients among different traits, stress tolerance index (STI), yield stability index (YSI) and combination of significant indices (CSI) of synthetic hexaploid wheat derived lines evaluated under normal (above diagonal) and water-stressed (below diagonal) environments.

Traits	DHE	DF	RWC	PHT	AL	SL	PL	SD	YLD	SM	TGW	GS	BY	HI	He
DHE	1	0.86**	− 0.09^ns^	0.40**	0.13*	0.10^ns^	0.10^ns^	0.17**	0.02^ns^	− 0.01^ns^	0.04^ns^	− 0.01^ns^	0.09^ns^	− 0.09^ns^	− 0.30**
DF	0.91**	1	− 0.10^ns^	0.29**	0.07^ns^	0.11^ns^	0.11^ns^	0.16*	− 0.03^ns^	− 0.01^ns^	− 0.04^ns^	0.06^ns^	0.06^ns^	− 0.10^ns^	− 0.29**
RWC	− 0.05^ns^	− 0.05^ns^	1	− 0.05^ns^	− 0.12^ns^	− 0.05^ns^	− 0.08^ns^	− 0.07^ns^	− 0.05^ns^	0.08^ns^	0.03^ns^	− 0.06^ns^	− 0.16*	0.09^ns^	0.11^ns^
PHT	0.48**	0.48**	− 0.02^ns^	1	− 0.08^ns^	0.17*	0.44**	0.34**	0.04^ns^	− 0.08^ns^	0.33**	0.00^ns^	0.22**	− 0.02^ns^	− 0.07^ns^
AL	0.04^ns^	− 0.02^ns^	− 0.18*	− 0.09^ns^	1	0.33**	0.23**	0.11^ns^	0.18**	0.14*	− 0.04^ns^	0.03^ns^	0.18**	0.07^ns^	− 0.03^ns^
SL	0.10^ns^	0.07^ns^	− 0.25**	0.16*	0.38**	1	0.41**	0.54**	0.25**	− 0.03^ns^	0.01^ns^	0.29**	0.32**	− 0.08^ns^	− 0.27**
PL	0.06^ns^	0.09^ns^	− 0.04^ns^	0.39**	0.20**	0.35**	1	0.55**	0.21**	0.05^ns^	0.11^ns^	0.15^ns^	0.22**	0.13^ns^	− 0.14*
SD	0.12^ns^	0.14*	− 0.12^ns^	0.24**	0.06^ns^	0.47**	0.47**	1	0.18**	− 0.10^ns^	0.19**	0.13^ns^	0.18*	0.09^ns^	− 0.28**
YLD	− 0.20^ns^	− 0.19^ns^	− 0.06^ns^	− 0.08^ns^	− 0.03^ns^	− 0.04^ns^	0.00^ns^	− 0.03^ns^	1	0.38**	0.12^ns^	0.38**	0.58**	0.73**	0.03^ns^
SM	0.05^ns^	0.03ns	0.11^ns^	0.01^ns^	0.13^ns^	− 0.05^ns^	0.11^ns^	− 0.08^ns^	0.44**	1	− 0.22**	− 0.18^ns^	0.15*	0.32**	− 0.01^ns^
TGW	− 0.28**	− 0.26**	0.11^ns^	0.02^ns^	− 0.26**	− 0.24**	− 0.14^ns^	− 0.07^ns^	0.09^ns^	− 0.31*	1	− 0.28**	0.10^ns^	0.12^ns^	0.16*
GS	− 0.00^ns^	− 0.00^ns^	− 0.26**	− 0.06^ns^	0.12^ns^	0.28**	0.01^ns^	0.09^ns^	0.28**	− 0.13^ns^	0.37**	1	0.27**	0.26**	− 0.01^ns^
BY	0.03^ns^	0.04^ns^	0.04^ns^	0.26**	− 0.08^ns^	0.05^ns^	0.08^ns^	0.03^ns^	0.47**	0.35**	− 0.00^ns^	0.18*	1	− 0.03^ns^	− 0.10^ns^
HI	− 0.22**	− 0.22**	− 0.13^ns^	− 0.25**	0.01^ns^	− 0.04^ns^	− 0.00^ns^	− 0.05^ns^	0.69**	0.19**	0.08^ns^	0.18*	− 0.23**	1	0.11^ns^
He	− 0.41**	0.43**	0.10^ns^	− 0.20**	− 0.11^ns^	− 0.36**	− 0.26**	− 0.36**	0.21**	− 0.13^ns^	0.55**	− 0.15*	− 0.06^ns^	0.22**	1
STI	− 0.15*	− 0.14^ns^	− 0.06^ns^	− 0.02^ns^	− 0.00^ns^	0.07^ns^	0.10^ns^	0.08^ns^	0.87**	0.37**	0.06^ns^	0.37**	0.13^ns^	0.45**	0.54**
YSI	− 0.17*	− 0.17*	− 0.02^ns^	− 0.13^ns^	− 0.08^ns^	− 0.20**	− 0.15*	− 0.12^ns^	0.57**	0.27**	0.09^ns^	− 0.04^ns^	0.22**	0.21**	0.48**
CSI	− 0.14^ns^	− 0.13^ns^	− 0.06^ns^	− 0.02^ns^	0.01^ns^	0.07^ns^	0.10^ns^	0.08^ns^	0.87**	0.38**	0.05^ns^	0.37**	0.12^ns^	0.44**	0.55**

DHE, days to heading; DF, days to flowering; RWC (%), relative water content; PHT (cm), plant height; AL (cm), awn length; SL (cm), spike length; PL (cm), peduncle length; SD, stem diameter (mm); YLD (g/m^2^), grain yield; SM, spike per m^2^; TGW (g), thousand-grain weight; GS, grains per spike; He, hectoliter (kg/he); BY (g/m^2^), biological yield; HI (%), harvest index; STI, stress tolerance index; YSI, yield stability index; CSI, combination of significant indices.

ns; * and **Non-significant, significant at 0.05 and 0.01 probability level, respectively.

**Figure 2:**
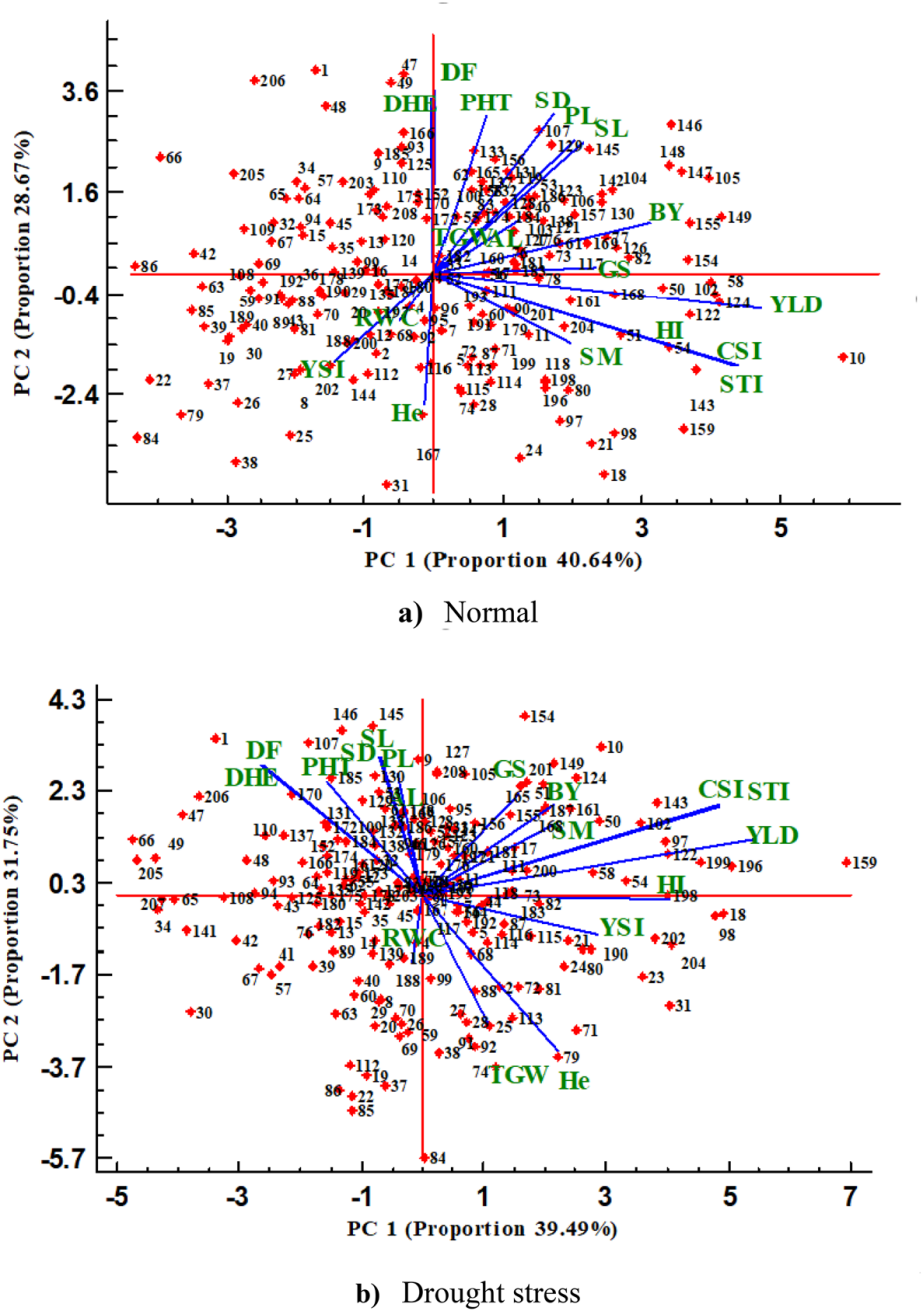
Principal component analysis of traits measured in the synthetic hexaploid wheat derived lines under normal (**a**) and water stress (**b**) conditions. Horizontal and vertical axes are the first and second principal components, respectively. DHE, days to heading; DF, days to flowering; RWC (%), relative water content; PHT (cm), plant height; AL (cm), awn length; SL (cm), spike length; PL (cm), peduncle length; SD, stem diameter (mm); YLD (g/m^2^), grain yield; SM, spike per m^2^; TGW (g), thousand-grain weight; GS, grains per spike; He, hectoliter (kg/he); BY (g/m^2^), biological yield; HI (%), harvest index; STI, stress tolerance index; YSI, yield stability index; CSI, combination of significant indices. The Number of genotypes is according to the first column (Genotype code) in table S3.

Principal component analysis (PCA) based on agro-morphological, physiological, and drought tolerance indices was performed (Fig. 2a, b). The principal component graph revealed that the first two components explained 69.31% and 71.24% of trait variation at normal irrigation and water stress conditions, respectively (Fig. 2a, b). The graphs revealed that phenological traits (DHE and DF) and traits related to plant morphology and architecture (PHT, AL, SL, and PL) were strongly and positively associated with each other under both moisture conditions and were grouped together. Also, STI, YSI, and CSI had a positive relationship with He in both water conditions. At normal irrigation, the first PC (PC1) had higher correlation with yield and its component and could be considered as “yield potential” factor (Fig. 2a). The second PC (PC2) had a positive correlation with phenological and vegetative traits, and was named “vegetative” factor (Fig. 2a). Genotypes 10, 50, 51, 54, 58, 102, 122, 124, 154, 155, 159, and 168 had the highest values for yield and its component. Under water stress conditions, PC1 had a higher correlation with yield and stress tolerance indices and was named “yield potential and drought tolerance” factor (Fig. 2b). At this conditions, PC2 had a positive correlation with yield components such as GS and SM which was named “yield component” factor (Fig. 2b). Selection based on high PC1 and PC2 may result in drought-tolerant genotypes and high potential for yield. As a result, genotypes 10, 18, 50, 54, 58, 97, 98, 102, 122, 124, 143, 149, 154,159, 196, 198, and 199 were found to have high yield potential.

### Interrelationships of traits (Step wise regression and path-coefficient analysis)

Based on the results of the stepwise regression analysis, spike m^−2^, grain spike^1^, thousand-grain weight, and awn length were the components that explained grain yield under normal water conditions (*P* < 0.01) (Table 4). These traits had significantly positive correlations with grain yield except for thousand-grain weight (Table 3). In water stress environments, the most important contributing traits to grain yield were spike m^−2^, thousand-grain weight, grain spike^−1^ and hectoliter (*P* < 0.01) (Table 4). Three of these traits were the same under both moisture regimes.

**Table 4:** Results of stepwise regression analysis for traits associated with grain yield in synthetic hexaploid wheat derived lines under normal and water stress conditions.

Variable	R^2^ partial	R^2^ model	SE	C(p)	b
**Normal**					
SM	0.1486	0.1486	8.41	148.61	0.57
GS	0.2154	0.3640	0.06	65.44	0.61
TGW	0.1473	0.5113	1.65	9.22	0.42
AL	0.0154	0.5266	1.36	5.14	0.12
**Drought stress**					
SM	0.1953	0.1953	0.05	139.44	0.68
TGW	0.1229	0.3182	1.59	91.42	0.44
GS	0.2055	0.5237	1.04	9.82	0.57
He	0.0163	0.5400	1.35	5.17	0.15

SM, spike per m^2^; TGW (g), thousand-grain weight; GS, grains per spike; AL, awn length; He, hectoliter (kg/he); SE, standard error; R^2^, the coefficient of determination showing the contribution of each trait to grain yield variation; C(p), Mallow’s cp statistic; b, coefficient of regression.

Based on path analysis, the correlation coefficients were partitioned according to their direct and indirect effects on grain yield (Table 5). Under normal water environments, grain spike^−1^ (0.61), spike m^−2^ (0.57), and thousand-grain weight (0.42) had the strongest direct effect on grain yield (Table 4). Also, spike m^−2^ (0.68), grain spike^−1^ (0.57) and thousand-grain weight (0.44) had strong and direct effects on grain yield, explaining 54% of the total yield variation under water stress environments (Table 4). Under normal water conditions, spike m^−2^ indirectly and negatively affected grain yield through thousand-grain weight (Table 5). Under water stress conditions, grain spike^−1^ indirectly and negatively affected grain yield through thousand-grain weight (Table 5).

**Table 5:** Path coefficient analysis with respect to the effect of four wheat traits on grain yield tested under normal and water stress conditions over 2 years.

Traits	X1	X2	X3	X4	Correlation with yield
**Normal**					
SM (X1)	**0.57**	− 0.10	− 0.18	0.12	0.38**
GS (X2)	− 0.11	**0.61**	0.17	0.02	0.38**
TGW (X3)	− 0.09	0.11	**0.42**	− 0.02	0.12^ns^
AL (X4)	0.01	0.01	− 0.01	**0.12**	0.18**
**Water stressed**					
SM (X1)	**0.68**	− 0.21	− 0.09	− 0.08	0.44**
TGW (X2)	− 0.13	**0.44**	− 0.16	0.24	0.09^ns^
GS (X3)	− 0.07	− 0.21	**0.57**	− 0.08	0.28**
He (X4)	− 0.02	0.08	− 0.02	**0.15**	0.21**

ns; **Non-significant and significant at 0.01 probability level, respectively.

Bold numbers on diagonal denote direct effects, out diagonal numbers indicate indirect effects of traits.

Significant values are in underline.

AL (cm), awn length; YLD (g/m^2^), grain yield; SM, spike per m^2^; TGW (g), thousand-grain weight; GS, grains per spike; He, hectoliter (kg/he).

### Identification of high yielding SHW-DL

**Figure 3:**
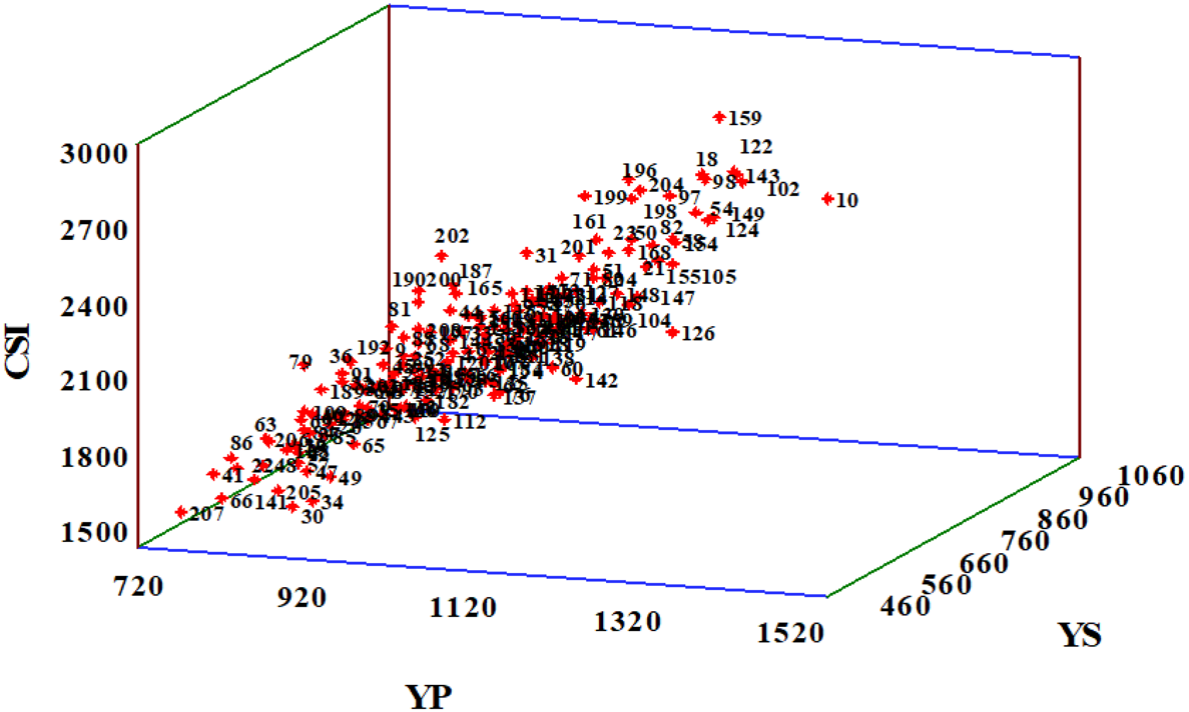
3‐plot among yield in normal condition (Yp), yield in stress condition (Ys) and combination of significant indices (CSI) for 184 synthetic hexaploid wheat and 8 common wheats. The Number of genotypes is according to the first column (Genotype code) in table S3.

High-yielding SHW-DL were identified using averaged data across two growing seasons under the two irrigations regime (Table S2). The top ten high-yielding lines of SHW-DL were recognized under normal and water stress conditions. Based on field performance data, genotypes 18, 98, 102, 122, 143, and 149 had high yield under normal and water stress environments with high yield components (GS, TGW, and SM). Based on the PCs, genotypes 10, 50, 51, 54, 58, 102, 122, 124, 154, 155, 159, and 168 were recognized for high yield and yield components at normal water conditions. Genotypes 10, 18, 50, 54, 58, 97, 98, 102, 122, 124, 143, 149, 154,159, 196, 198, and 199 were found to have a high potential for yield under water stress conditions. Based on the 3‐plot of yield in normal water conditions (Yp), yield in water stress conditions (Ys), and combination of significant indices (CSI), the best genotypes were 10, 18, 54, 82, 97, 98, 102, 122, 124, 143, 149, 154, 159, 196, 198, and 204. These genotypes had high productivity and stability in drought-stressed conditions (Fig. 3). Overall, using different methods, genotypes 10, 54, 102, 122, 124, 143, 149, 154, 159, and 198 were identified as the best performers under the tested environments.

## Discussion

Despite developing several synthetic hexaploid kinds of wheat, little is known about the adaptation and capability of selection for drought tolerance in dry regions. To the best of our knowledge, this is the first study evaluating a diverse panel of 184 SHW-DL in Iran aiming to obtain robust trait value data for wheat improvement under drought conditions. The results of this project can be helpful for further improvement of drought tolerance traits of different wheat varieties. The high genetic variation observed for several drought tolerance-related traits can facilitate the selection of drought tolerance genotypes for arid environments. The significant interaction of genotypes and irrigation regimes revealed the genetic values of SHW-DL compared to common wheat for the intended purpose.

As expected, water stress affected several measured traits and caused significant reductions in BY, RWC, PHT, HI, PL, SD, He, grain yield, and its components (SM and TGW). Despite the reduction in traits related to productivity, response to water stress varied within the germplasm, indicating enough diversity for selection and improvement during the breeding program. This was vividly reflected by high genotypic coefficient of variation (GCV) for most measured traits especially peduncle length (19.32), awn length (17.7), grain spike^−1^ (16.03), and grain yield (13.41). These findings agree with results obtained by Al-tabbal and Al-fraihat^31^. In line with Blum^17^ and Ebrahimiyan et al.^18^ the GCV for most traits including yield was decreased due to water stress. Higher genetic variation coupled with a high heritability estimation enormously benefits phenotypic selection. According to Dabholkar^32^ who defined the magnitude of heritability estimates as low (5–10%), medium (10–30%), and high (> 30%), the heritability of significant variables were considered high in the current research. In this study, the heritability estimate was high for DHE, DF, PHT, AL, SL, PL, SD, YLD, TGW, GS, and He. High heritability was reported for grain yield, the number of seeds per spike, plant height, heading date and thousand grain weight^31^.

Evaluation of the SHW-DL in the two consecutive growing seasons revealed that over 31% grain yield loss was incurred by water stress treatment compared to the normal conditions, which is lower than the loss reported for common wheat (36%) by others^33^. Recently, a much lower yield decrease (25%) in SHW-DL than bread wheat (47%) was reported due to water stress which reveals the higher potential of SHW-DL to retain the amount of leaf chlorophyll under drought^14^. Our findings were inconstant with previous research that revealed the superiority of wheat lines derived from synthetic hexaploid wheats (SHWs) as compared to conventional bread wheats using different morphological and physiological traits under control and osmotic stress^34^. This may be attributed to the possibility of quicker relocation of metabolites from leaves and stems to developing grain in SHW-derived lines under drought stress^35^.

Direct selection based on grain yield is mainly practiced in wheat breeding programs. However, the presence of genotype × environment interactions and low heritability reduce selection efficiency of using grain yield as the sole criterion. To overcome these difficulties, breeders are focused on other traits that can be used in parallel or independently of yield in a multi traits approach. Therefore, indirect selection can be achieved for both conditions by determining the relationship between yield and its components. In our study, the relationship between grain yield, spike length, grain spike^−1^, biological yield, and harvest index was significantly positive, which is in agreement with the previous findings^36–39^. The direct effects of traits were recognized using the stepwise regression method for both water environments, including grain spike^−1^ and spike m^−2^ indicating they are the most reliable ones to improve the selection of high-yield lines. Kumar et al.^40^ also concluded that the number of grain spike^−1^ and harvest index directly positively affected grain yield. According to path analysis, the indirect effects of these traits were also revealed, in which TGW was the one with the most considerable indirect effect in normal, and water-stressed conditions. Blum^17^ reported that indirect selection via yield components and other traits could be more efficient than direct selection if the traits are related to yield and have a higher heritability than yield. The selection-based index is another complex approach but can avoid the limits of single-trait selection. The selection-based index approach targets the simultaneous improvement of several traits, including the grain yield^41, 42^. Based on the PCs, the results also showed that three tolerant indices of STI, YSI, and CSI positively correlated with yield, yield component (SM and GS), BY, HI, and He under both moisture environments. Therefore, these indices can help distinguish genotypes with different levels of drought tolerance.

Overall, drought-tolerant genotypes were identified using morphological, RWC, and stress tolerance indices (STI, YSI, and CSI). In this regard, genotypes 10, 54, 102, 122, 124, 143, 149, 154, 159, and 198 were introduced as superior genotypes using grain yield, multivariate analysis, and drought tolerance indices among the 184 synthetic hexaploid derived lines. A combination of significant indices (CSI), creating a composite index (including MP, HM, GMP, and STI), provided a suitable criterion to identify drought tolerant lines in this study which is in agreement with the previous study^29^.

## Conclusion

To the best of our knowledge, this is the first study evaluating a diverse panel of hexaploid synthetic wheat in the semi-arid regions of central Iran. The results indicated that the 184 SHW-DL studied constitutes functional genetic variability for morphological, productivity, and drought tolerance traits. The results also suggested a considerable potential for wheat breeding to face global climate changes, especially in arid environments. SHW-derived lines' superiority over common wheat was mainly due to early maturity, dwarfing, higher thousand-grain weight, peduncle length, stem diameter, and harvest index. Using multi approach genetic analysis, several SHW-DL with high production and resistance to water stress were identified in the present study. Our results also indicated that the priority of traits entering to regression model (for predicting grain yield) was not the same under normal and water stress conditions suggesting different strategies in indirect selection programs.

## Materials and methods

### Plant material

In this study, 184 spring SHW-derived lines were chosen from the collection available at the gene bank at CIMMYT (Table S3 in supplementary file). Along with these plant materials, eight bread wheat cultivars were used as control including AAC Scotia, Carbery, Norwell, Sable (from Canada), Pishtaz, Qhods, Kavir, and Roshan from Iran. Each SHW-DL was developed by crossing an *Ae. tauschii* accession with a modern tetraploid wheat genotype. All of the individual synthetic lines in the panel are derived from at least one cross to elite hexaploid wheat, resulting in different degrees of synthetic genetic materials in the resulting lines, ranging from 2nd degree synthetic (primary SHW crossed to one common hexaploid line) to 5th degree synthetic (primary synthetic crossed to four common hexaploid lines). Supplementary Table S3 lists the details of the germplasm used in the study. There are 19 distinct *Ae. tauschii* and 13 distinct tetraploid accessions used in the initial crosses contribute to 23 distinct primary SHWs (Table S4). Our plant material is a public panel and complies with relevant institutional, national, and international guidelines and legislation.

### Field trials and data collections

#### Study area

Field experiments were conducted during two winter cropping seasons (October to June of 2018–2019 and 2019–2020) at the Research Farm of Isfahan University of Technology, located in Lavark, Najaf Abad, Iran (32° 30́ N, 51° 20́ E). Based on annual averages of long-term climatic data, the mean annual temperature and precipitation of the station were 14.5 °C and 140 mm, respectively. The trend of temperature and humidity of Najaf Abad during the growing season (Oct–Jun) of 2018–2019 and 2019–2020 is depicted in Fig S2. The site has silty clay loam soil with pH = 8.3. The soil was calcareous, containing 390 g kg^–1^Ca-carbonate equivalent, 4.0 g kg^–1^ organic C, and 0.77 g kg^–1^ total N. The soil was non-saline and non-sodic. The analysis of field soil related to two growing seasons (Oct–Jun), 2018–2019 and 2019–2020, are shown in Table S5.

#### Field experiment

Each plot contained four planting rows (2 m long, with 20 cm spacing between the rows) and approximately 300 plant/m^2^ density. The experiments were laid out according to a balanced 14 × 14 simple lattice square design under two water regimes (normal and water-stressed) in both consecutive years. Water stress treatment was applied at the 50% flowering stage through irrigation when 85–90% of water at field capacity (FC) was depleted from the root zone. For normal water conditions, irrigation was continued at 45–50% of FC until maturity^43^. Soil moisture was measured by standard gravimetric methods^44^ at depths of 0–20, 20–40 and 40–60 cm using auger. The irrigation depth was determined according to the following equation:

$${\text{I}} = \left[ {\left( {{\text{FC}} -\uptheta } \right)/{1}00} \right]\,{\text{D}} \times {\text{B}}$$

where I is the irrigation depth (cm), FC is the soil gravimetric moisture percent at field capacity, θ is the soil gravimetric moisture percent at irrigating time, D is the root zone depth (60 cm), and B is the soil bulk density at root zone (1.4 g/cm^3^). The water was applied using a basin irrigation system. The water volume for each treatment was measured by a volumetric counter. The depth of irrigation (Ig) was calculated according to the following equation:

$${\text{Ig}} = {\text{I}} \times {1}00/{\text{Ea}}$$

where I is the irrigation depth and Ea is the irrigation efficiency (%) assumed as 75% during the growing season.

### Data collection

Leaf relative water content (RWC) was measured 10–12 days after implementing the water stress treatment using the method described by Barrs and Weatherley (1)^45^:

1 $${\text{RWC}}\,{\text{(\% )}} = ({\text{FW }}{-}{\text{ DW}}) \times {1}00/({\text{TW }}{-}{\text{ DW}})$$

where FW, DW and TW are the fresh weight, dry weight and turgid weight, respectively.

Days to heading (DHE) was the period between sowing and when 50% of the spikes emerged from the leaf sheath. Days to flowering (DF) were recorded as the number of days after seeding to when 50% of the plants within a plot were at the pollination stage. Plant height (PHT), awn length (AL), spike length (SL), peduncle length (PL), stem diameter (SD), grains spike^−1^ (GS), spike m^−2^ (SM), thousand-grain weight (TGW), and grain yield (YLD), hectoliter (He), biological yield (BY) and harvest index (HI) were measured. Hectoliter weight was measured using a glass graduated cylinder (100 cm^3^).

### Statistical analyses

The normal distribution of the residues was verified with the Smirnov–Kolmogorov normality test before undergoing the ANOVA analysis using SAS 9.4 software^46^. No transformation of data was necessary to achieve normal distribution. Analysis of variance (ANOVA) was conducted to partition the total variation into components based on the expected mean square in the software of SAS 9.4^46^ using the general linear model (GLM). Least significant difference (LSD) values were calculated at the 5% probability level when the F-value was significant. Pearson correlation coefficients among the studied traits were calculated separately for the two water regimes using PROC CORR in SAS software. Principal component analyses (PCA) were performed based on a correlation matrix constructed over the mean data of all replicates. PCA bi-plots were plotted separately for the stressed and normal water conditions using Statgraphics software to show the relationships among studied genotypes based on recorded traits. The genetic coefficient of variation (GCV) was calculated as:

$${\text{GCV}} = (\upsigma _{{\text{g}}} /\upmu )*{1}00$$

where σ_g_ is the square root of the genotypic variance, and µ is the phenotypic mean^47^. Estimations of heritability were based on the expected mean squares of ANOVA with the following Eq. (2)^48^:

2 $${\text{h}}^{{2}} = \sigma_{{\text{g}}}^{2} /\left( {\sigma_{{\text{g}}}^{2} + \sigma_{{\text{e}}}^{2} /{\text{r}}} \right)$$

σ_g_^2^, σ_e_^2^ variance component of genetic and error, respectively.

Stepwise regression analysis was performed to identify the most important traits involved to grain yield variation. Path coefficients analysis was done using correlation coefficients with respect to grain yield as dependent variable and the selected traits in stepwise characters as independent effects using SAS software^46^. To select for high yielding genotypes under normal and water stressed conditions stress tolerance index (STI), yield stability index (YSI) and combination of significant indices (CSI) was calculated using the following formula (3,4) according to Fernandez^20^:

3 $${\text{STI}} = ({\text{Yp}} * {\text{Ys}})/\left( {{\text{Xp}}} \right)^{{2}} ;$$

4 $${\text{YSI}} = {\text{Ys}}/{\text{Yp}};$$

5 $${\text{CSI}}_{{\text{i}}} = {1}/{2 }[({\text{r}}_{{{\text{YP}}.{\text{MP}}}} \times {\text{ MP}}_{{\text{i}}} ) + ({\text{r}}_{{{\text{YP}}.{\text{GMP}}}} \times {\text{ GMP}}_{{\text{i}}} ) + {\text{(r}}_{{{\text{YP}}.{\text{HM}}}} \times {\text{ HM}}_{{\text{i}}} {)} + {\text{(r}}_{{{\text{YP}}.{\text{STI}}}} \times {\text{ STI}}_{{\text{i}}} {)} + ({\text{r}}_{{{\text{YS}}.{\text{MP}}}} \times {\text{ MP}}_{{\text{i}}} ) + ({\text{r}}_{{{\text{YS}}.{\text{GMP}}}} \times {\text{ GMP}}_{{\text{i}}} ) + ({\text{r}}_{{{\text{YS}}.{\text{HM}}}} \times {\text{ HM}}_{{\text{i}}} ) + ({\text{r}}_{{{\text{YS}}.{\text{STI}}}} \times {\text{ STI}}_{{\text{i}}} )$$

where Ys is grain yield of a test genotype under water stressed conditions; Yp is grain yield of a test genotype under normal conditions, and Xp is mean yield of test genotypes under normal conditions.

## Supplementary Information

Supplementary Information.

## Data Availability

The datasets analyzed during the current study are available from the corresponding author on reasonable request.
