# Supplementary material for: Potentials of synthetic hexaploid wheats to improve drought tolerance
Source: Sci Rep. 2022 Nov 28;12:20482. doi: 10.1038/s41598-022-24678-5 (PMC9705419; doi:10.1038/s41598-022-24678-5)
Supplement: Supplementary file 1 — Supplementary Information. [file 41598_2022_24678_MOESM1_ESM.docx]

| **Table S1.** Analysis of variance for morphological, phenological and physiological traits tested under well-watered and drought stressed conditions in synthetic hexaploid wheats during two years | | | | | | | | | | |
| --- | --- | --- | --- | --- | --- | --- | --- | --- | --- | --- |
| **SV** | **df** | **Mean Square** | | | | | | | | |
|  |  | **DHE** | **DF** | **RWC** | **PHT** | **AL** | **SL** | **PL** | **SD** | **YLD** |
| **Irrigation (I)** | 1 | 11.66 ^ns^ | 37846.55** | 119077.63** | 957.14 ^ns^ | 0.72 ^ns^ | 0.29 ^ns^ | 111.46* | 173.59 ** | 36567453.03 ** |
| **Year (Y)** | 1 | 2983.31** | 21432.51** | 6350.72 ^ns^ | 20811.79** | 48.62** | 164.40** | 2660.89** | 30.85 ** | 72333259.81** |
| **Y * I** | 1 | 124.46* | 34955.83** | 15154.62 ^ns^ | 2507.36** | 2.25 ^ns^ | 0.33 ^ns^ | 162.17* | 2.50 ^ns^ | 570439.08 ^ns^ |
| **Rep (Y * I)** | 8 | 13.89* | 14.70** | 3929.07** | 258.88** | 0.54 ^ns^ | 2.25** | 10.78 ^ns^ | 0.44 ** | 1375968.43** |
| **Genotype (G)** | 191 | 52.73** | 29.58** | 95.54** | 398.73** | 4.31** | 7.51** | 76.30** | 0.46 ** | 71963.21** |
| **Common wheats** | 7 | ** | ** | ** | ** | ** | ** | ** | ** | * |
| **Synthetic wheats** | 183 | ** | ** | ** | ** | ** | ** | ** | ** | ** |
| **Com vs. Syn** | 1 | ** | * | ^ns^ | ** | ^ns^ | ^ns^ | ^ns^ | ** | ^ns^ |
| **G * I** | 191 | 6.61* | 3.91* | 79.55 ** | 45.00 ^ns^ | 0.37 ^ns^ | 0.50 ^ns^ | 7.28 ^ns^ | 0.06 ** | 26774.36 ^ns^ |
| **G * Y** | 191 | 12.78** | 7.29** | 75.63 ** | 77.10** | 0.68** | 1.15** | 9.65** | 0.07 ** | 50992.67** |
| **G * Y * I** | 191 | 6.78* | 3.22 ^ns^ | 84.58** | 40.55 ^ns^ | 0.27 ^ns^ | 0.51 ^ns^ | 7.62 ^ns^ | 0.08 ** | 22354.38 ^ns^ |
| **Error** | 763 | 5.46 | 3.19 | 56 | 45.48 | 0.32 | 0.52 | 6.77 | 32.30 | 25555.8 |
| **R^2^** |  | 0.81 | 0.97 | 0.84 | 0.81 | 0.83 | 0.83 | 0.81 | 0.91 | 0.88 |
| **CV (%)** |  | 1.33 | 0.93 | 10.60 | 5.80 | 9.82 | 6.22 | 11.99 | 6.72 | 19.09 |

| **Table S1.** continued | | | | | | | |
| --- | --- | --- | --- | --- | --- | --- | --- |
| **SV** | **df** | **Mean Square** | | | | | |
|  |  | **SM** | **TGW** | **GS** | **He** | **BY** | **HI** |
| **Irrigation (I)** | 1 | 720595.60 ** | 51527.47** | 1096.71 * | 21465.70** | 33117754* | 35726.48* |
| **Year (Y)** | 1 | 18634712** | 53.25 ^ns^ | 16240.52** | 890.33 ^ns^ | 1072644207** | 45715.35* |
| **Y * I** | 1 | 753791.40** | 3888.09** | 9.58 ^ns^ | 6.87 ^ns^ | 2386709 ^ns^ | 2499.53 ^ns^ |
| **Rep (Y * I)** | 8 | 1975.60 ^ns^ | 68.40** | 76.27 * | 205.32** | 1768716** | 2784.03** |
| **Genotype (G)** | 191 | 57185.10 ** | 105.58** | 160.94 ** | 70.80** | 190419** | 120.39** |
| **Common wheats** | 7 | ** | ** | ** | ** | ^ns^ | ^ns^ |
| **Synthetic wheats** | 183 | ** | ** | ** | ** | ** | ** |
| **Com vs. Syn** | 1 | ^ns^ | ** | ^ns^ | * | ^ns^ | ** |
| **G * I** | 191 | 24640.20 ^ns^ | 28.07** | 43.79 ** | 23.73** | 106321 ^ns^ | 78.07 ^ns^ |
| **G * Y** | 191 | 17404.40 ^ns^ | 23.94* | 12.22 ^ns^ | 17.23 ^ns^ | 132128 ^ns^ | 87.09 ^ns^ |
| **G * Y * I** | 191 | 11378.7 ^ns^ | 22.66 ^ns^ | 9.45 ^ns^ | 13.76 ^ns^ | 95126 ^ns^ | 75.73 ^ns^ |
| **Error** | 763 | 24376.5 | 19.57 | 30.62 | 14.90 | 113596 | 74.87 |
| **R^2^** |  | 0.91 | 0.86 | 0.72 | 0.81 | 0.93 | 0.74 |
| **CV (%)** |  | 24.03 | 12.70 | 14.81 | 4.82 | 15.35 | 21.52 |
| ns; *; ** Non-significant, Significant at 0.05 and 0.01 probability level, respectively  DHE days to heading, DF days to flowering, RWC (%) relative water content, PHT (cm) plant height, AL (cm) awn length, SL (cm) spike length, PL (cm) peduncle length, SD stem diameter (mm), YLD (g/m^2^) grain yield, SM spike per m^2^, TGW (g) thousand-grain weight, GS grains per spike, He hectoliter (kg/m^3^), BY (g/m^2^) biological yield, HI (%) harvest index, R^2^ coefficient of determination, CV (%)coefficient of variation. | | | | | | | |

| **Table S2.** Six of the important traits mean belonged to SHW population and bread wheat under well-irrigated and drought stressed conditions. | | | | | | | | | | | | |
| --- | --- | --- | --- | --- | --- | --- | --- | --- | --- | --- | --- | --- |
| **Genotype code** | **DHE** | | **PHT** | | **YLD** | | **SM** | | **TGW** | | **GS** | |
|  | **Irrrigated** | **Drought stress** | **Irrrigated** | **Drought stress** | **Irrrigated** | **Drought stress** | **Irrrigated** | **Drought stress** | **Irrrigated** | **Drought stress** | **Irrrigated** | **Drought stress** |
| 1 | 182.50 | 183.50 | 136.72 | 136.50 | 847.59 | 613.48 | 638.76 | 621.84 | 38.48 | 25.95 | 35.45 | 43.53 |
| 2 | 172.25 | 173.00 | 114.75 | 113.50 | 974.18 | 731.36 | 749.72 | 638.37 | 36.24 | 30.24 | 31.11 | 36.04 |
| 4 | 177.00 | 175.25 | 122.50 | 124.00 | 974.34 | 668.26 | 942.01 | 740.00 | 30.40 | 27.56 | 34.12 | 33.04 |
| 5 | 178.25 | 179.00 | 113.42 | 114.62 | 1048.46 | 730.86 | 849.62 | 730.71 | 35.80 | 30.93 | 32.68 | 28.57 |
| 7 | 176.25 | 178.75 | 107.25 | 113.56 | 1025.55 | 722.83 | 776.81 | 704.85 | 35.83 | 27.92 | 39.84 | 34.20 |
| 8 | 175.75 | 178.00 | 99.75 | 100.75 | 957.61 | 647.79 | 552.50 | 463.75 | 34.12 | 28.75 | 40.59 | 40.95 |
| 9 | 176.50 | 179.00 | 116.66 | 120.12 | 866.34 | 722.82 | 529.23 | 762.70 | 38.16 | 24.63 | 37.42 | 40.75 |
| 10 | 169.50 | 173.00 | 108.18 | 118.00 | 1374.28 | 789.16 | 810.17 | 719.22 | 37.82 | 26.24 | 40.88 | 44.70 |
| 11 | 173.50 | 173.50 | 110.50 | 114.50 | 1105.82 | 669.93 | 753.05 | 664.09 | 36.37 | 25.25 | 38.38 | 42.75 |
| 12 | 172.00 | 175.00 | 104.25 | 115.62 | 951.23 | 595.99 | 655.08 | 627.44 | 37.32 | 23.99 | 37.56 | 46.32 |
| 13 | 172.00 | 173.50 | 113.00 | 111.37 | 946.44 | 590.57 | 545.06 | 535.87 | 38.27 | 28.77 | 32.92 | 41.13 |
| 14 | 172.25 | 175.50 | 115.00 | 114.12 | 933.42 | 655.52 | 519.12 | 566.19 | 35.18 | 26.44 | 42.36 | 31.67 |
| 15 | 175.50 | 174.25 | 112.12 | 114.75 | 873.54 | 596.22 | 607.42 | 684.38 | 32.08 | 24.31 | 41.34 | 34.06 |
| 16 | 173.50 | 174.75 | 110.25 | 113.62 | 1006.28 | 628.28 | 467.68 | 566.73 | 37.27 | 24.39 | 32.51 | 40.07 |
| 17 | 173.50 | 174.25 | 115.25 | 111.25 | 1021.57 | 756.85 | 652.17 | 737.35 | 36.68 | 24.41 | 39.38 | 40.77 |
| 18 | 171.00 | 172.50 | 106.37 | 106.12 | 1165.42 | 901.44 | 619.87 | 509.24 | 40.59 | 31.37 | 44.94 | 49.79 |
| 19 | 170.00 | 169.75 | 105.25 | 106.50 | 828.14 | 570.90 | 441.27 | 561.16 | 40.23 | 32.64 | 37.94 | 37.38 |
| 20 | 169.50 | 172.25 | 121.75 | 116.75 | 857.51 | 595.83 | 629.62 | 568.33 | 39.96 | 38.97 | 32.52 | 33.65 |
| 21 | 169.00 | 171.00 | 113.87 | 116.00 | 1173.55 | 746.00 | 785.78 | 552.75 | 42.16 | 33.27 | 34.94 | 43.65 |
| 22 | 170.00 | 170.50 | 106.75 | 110.25 | 764.01 | 556.20 | 602.70 | 562.81 | 39.79 | 34.88 | 29.83 | 33.15 |
| 23 | 169.50 | 170.50 | 103.25 | 103.00 | 1102.12 | 793.54 | 791.42 | 617.14 | 38.71 | 30.72 | 38.23 | 40.45 |
| 24 | 169.75 | 172.50 | 110.62 | 111.75 | 1124.10 | 745.28 | 716.89 | 696.61 | 38.99 | 31.09 | 36.84 | 37.64 |
| 25 | 171.25 | 172.50 | 105.50 | 104.50 | 900.87 | 694.63 | 601.72 | 558.77 | 40.28 | 29.16 | 36.23 | 38.17 |
| 26 | 170.50 | 170.50 | 102.12 | 110.00 | 859.98 | 607.86 | 656.01 | 631.97 | 39.61 | 29.50 | 32.22 | 35.29 |
| 27 | 171.25 | 170.75 | 109.50 | 111.75 | 904.12 | 661.83 | 463.83 | 581.95 | 38.04 | 26.44 | 44.17 | 38.86 |
| 28 | 171.00 | 171.75 | 106.75 | 105.62 | 1086.34 | 649.33 | 838.15 | 616.33 | 39.21 | 31.31 | 34.25 | 31.19 |
| 29 | 175.25 | 175.00 | 117.75 | 114.50 | 946.47 | 638.44 | 674.03 | 606.58 | 39.84 | 32.52 | 36.29 | 31.64 |
| 30 | 175.25 | 173.75 | 110.00 | 108.12 | 877.05 | 460.91 | 789.85 | 413.60 | 36.46 | 27.83 | 30.26 | 42.42 |
| 31 | 171.00 | 170.25 | 100.75 | 102.00 | 981.18 | 836.96 | 765.80 | 759.19 | 39.43 | 32.54 | 35.33 | 36.63 |
| 32 | 177.25 | 175.75 | 108.00 | 115.25 | 832.78 | 678.98 | 524.76 | 665.52 | 34.76 | 23.18 | 44.29 | 40.40 |
| 33 | 174.50 | 174.75 | 117.50 | 106.87 | 965.02 | 709.22 | 772.47 | 717.34 | 36.34 | 22.47 | 38.42 | 45.66 |
| 34 | 177.25 | 176.50 | 109.50 | 104.75 | 901.00 | 462.90 | 678.21 | 551.15 | 37.13 | 22.51 | 37.39 | 39.86 |
| 35 | 172.75 | 173.50 | 113.37 | 114.25 | 899.70 | 607.50 | 675.89 | 821.88 | 36.26 | 21.58 | 37.00 | 34.97 |
| 36 | 174.00 | 172.75 | 109.37 | 111.62 | 824.42 | 716.83 | 542.87 | 651.93 | 42.51 | 26.55 | 35.46 | 41.14 |
| 37 | 171.00 | 172.50 | 106.50 | 103.87 | 835.30 | 588.92 | 586.15 | 655.68 | 40.47 | 35.04 | 31.88 | 35.37 |
| 38 | 170.00 | 172.25 | 99.62 | 107.25 | 887.19 | 641.04 | 723.22 | 629.59 | 39.31 | 35.34 | 30.06 | 35.53 |
| 39 | 170.50 | 172.75 | 117.00 | 119.25 | 825.97 | 573.68 | 597.66 | 740.04 | 45.86 | 33.30 | 22.08 | 32.43 |
| 40 | 171.00 | 172.50 | 119.00 | 118.75 | 824.91 | 623.20 | 638.67 | 669.94 | 45.89 | 37.20 | 29.92 | 31.52 |
| 41 | 177.00 | 175.25 | 122.25 | 122.12 | 730.71 | 560.91 | 522.44 | 592.44 | 38.59 | 28.94 | 31.89 | 37.34 |
| 42 | 179.50 | 178.00 | 121.00 | 125.00 | 837.61 | 558.29 | 634.86 | 568.92 | 37.87 | 27.27 | 31.68 | 37.72 |
| 43 | 174.50 | 175.25 | 127.25 | 125.75 | 926.62 | 587.72 | 732.90 | 676.93 | 39.59 | 27.78 | 26.31 | 31.31 |
| 44 | 176.00 | 175.75 | 132.25 | 126.75 | 923.52 | 762.51 | 912.45 | 627.65 | 39.48 | 31.47 | 33.09 | 35.02 |
| 45 | 176.25 | 176.25 | 135.25 | 127.50 | 878.31 | 687.60 | 603.41 | 601.67 | 44.10 | 32.34 | 36.37 | 34.33 |
| 46 | 177.00 | 175.25 | 129.50 | 123.00 | 1050.03 | 661.08 | 562.85 | 758.45 | 40.30 | 21.94 | 43.71 | 41.03 |
| 47 | 175.00 | 176.25 | 131.75 | 126.12 | 869.54 | 514.76 | 487.98 | 504.51 | 43.25 | 25.19 | 46.69 | 38.72 |
| 48 | 175.00 | 175.00 | 126.75 | 124.50 | 797.38 | 550.32 | 571.49 | 505.00 | 45.90 | 30.12 | 35.24 | 36.20 |
| 49 | 175.25 | 176.50 | 131.00 | 125.00 | 906.82 | 496.18 | 592.38 | 436.71 | 42.54 | 26.93 | 38.80 | 36.41 |
| 50 | 170.00 | 171.25 | 118.25 | 107.87 | 1127.81 | 803.71 | 846.02 | 852.23 | 37.55 | 25.46 | 42.19 | 36.53 |
| 51 | 170.50 | 171.00 | 111.50 | 114.50 | 1100.34 | 763.63 | 942.69 | 724.20 | 33.69 | 25.00 | 39.48 | 39.23 |
| 52 | 170.75 | 171.00 | 112.62 | 108.75 | 916.92 | 686.72 | 798.35 | 642.92 | 34.44 | 25.16 | 36.80 | 39.86 |
| 53 | 171.25 | 174.25 | 114.75 | 115.75 | 1051.58 | 646.15 | 534.91 | 645.40 | 36.17 | 22.82 | 36.28 | 43.24 |
| 54 | 173.25 | 175.00 | 114.25 | 110.50 | 1197.54 | 824.45 | 629.43 | 652.56 | 41.82 | 30.18 | 45.22 | 46.15 |
| 55 | 174.25 | 174.75 | 117.25 | 107.50 | 1017.41 | 615.69 | 585.07 | 537.17 | 34.83 | 24.48 | 38.14 | 40.32 |
| 56 | 173.75 | 175.00 | 114.00 | 111.25 | 1000.2 | 691.43 | 722.82 | 699.97 | 37.16 | 25.76 | 38.51 | 37.89 |
| 57 | 173.00 | 172.75 | 118.25 | 114.50 | 846.45 | 537.08 | 795.50 | 561.95 | 46.13 | 32.72 | 24.17 | 29.62 |
| 58 | 170.75 | 169.50 | 107.25 | 108.25 | 1185.95 | 782.78 | 898.49 | 495.09 | 36.33 | 26.36 | 38.43 | 43.96 |
| 59 | 175.50 | 173.25 | 111.50 | 110.25 | 863.45 | 654.39 | 591.97 | 475.20 | 42.09 | 33.71 | 33.48 | 38.32 |
| 60 | 175.00 | 174.25 | 118.00 | 114.75 | 1129.86 | 599.96 | 746.28 | 553.75 | 43.05 | 28.96 | 33.52 | 35.84 |
| 61 | 176.75 | 176.25 | 115.50 | 112.50 | 1143.88 | 654.87 | 862.60 | 639.89 | 40.57 | 23.29 | 35.36 | 44.08 |
| 62 | 177.25 | 175.00 | 122.00 | 122.87 | 1056.76 | 598.19 | 540.16 | 594.72 | 36.97 | 27.90 | 39.79 | 43.47 |
| 63 | 173.00 | 174.00 | 130.50 | 125.50 | 781.70 | 596.27 | 678.82 | 650.63 | 40.58 | 35.38 | 29.89 | 30.14 |
| 64 | 180.50 | 180.75 | 122.50 | 123.25 | 877.29 | 646.98 | 695.87 | 723.71 | 43.56 | 31.22 | 32.51 | 36.43 |
| 65 | 177.25 | 179.00 | 128.00 | 130.00 | 910.99 | 544.79 | 590.01 | 505.34 | 43.39 | 32.65 | 31.06 | 30.50 |
| 66 | 180.00 | 179.75 | 117.75 | 122.75 | 769.97 | 505.82 | 614.93 | 675.92 | 38.84 | 24.88 | 34.95 | 34.38 |
| 67 | 175.75 | 176.75 | 113.50 | 115.75 | 908.80 | 584.27 | 490.09 | 479.27 | 49.36 | 36.19 | 33.95 | 26.51 |
| 68 | 172.50 | 173.75 | 119.75 | 115.50 | 911.18 | 710.62 | 739.79 | 514.55 | 39.09 | 34.21 | 32.96 | 40.16 |
| 69 | 174.50 | 174.25 | 118.50 | 112.00 | 809.93 | 621.15 | 694.72 | 450.77 | 44.54 | 37.16 | 25.87 | 37.24 |
| 70 | 172.75 | 171.50 | 132.50 | 116.25 | 883.90 | 618.52 | 622.10 | 577.37 | 44.46 | 35.28 | 31.47 | 27.76 |
| 71 | 173.00 | 170.75 | 113.25 | 109.50 | 1058.56 | 766.99 | 641.01 | 481.61 | 50.57 | 41.22 | 31.94 | 34.62 |
| 72 | 172.75 | 171.25 | 118.25 | 119.75 | 1039.20 | 695.24 | 647.67 | 744.11 | 39.45 | 30.63 | 35.88 | 30.28 |
| 73 | 174.75 | 174.75 | 105.00 | 102.00 | 1052.64 | 748.53 | 595.96 | 621.51 | 38.51 | 28.03 | 45.19 | 39.54 |
| 74 | 171.00 | 171.25 | 104.00 | 100.75 | 1096.40 | 663.90 | 773.17 | 631.31 | 47.44 | 38.05 | 29.14 | 29.23 |
| 76 | 175.00 | 175.00 | 132.50 | 131.00 | 1077.21 | 576.66 | 597.37 | 518.81 | 45.85 | 31.50 | 38.48 | 32.66 |
| 77 | 175.25 | 176.00 | 122.87 | 124.00 | 1151.24 | 665.38 | 676.98 | 628.61 | 42.22 | 29.37 | 40.48 | 38.82 |
| 78 | 175.50 | 175.00 | 126.75 | 117.75 | 1079.14 | 666.62 | 652.90 | 639.06 | 46.22 | 28.05 | 36.46 | 43.65 |
| 79 | 169.50 | 170.50 | 115.00 | 113.75 | 753.69 | 743.50 | 595.23 | 610.85 | 41.34 | 32.21 | 30.99 | 36.87 |
| 80 | 170.00 | 170.00 | 109.25 | 106.75 | 1106.22 | 748.77 | 706.69 | 704.68 | 44.56 | 30.58 | 37.58 | 37.35 |
| 81 | 173.75 | 174.00 | 115.50 | 108.50 | 851.04 | 765.47 | 658.86 | 671.13 | 43.33 | 28.89 | 30.09 | 33.30 |
| 82 | 176.25 | 176.50 | 119.00 | 121.00 | 1160.65 | 786.55 | 676.94 | 627.51 | 51.37 | 41.24 | 33.52 | 32.49 |
| 83 | 175.00 | 173.50 | 130.75 | 129.75 | 1010.22 | 676.68 | 594.76 | 508.47 | 42.05 | 32.93 | 36.13 | 39.14 |
| 84 | 172.50 | 170.75 | 94.25 | 98.25 | 824.20 | 599.11 | 497.22 | 411.17 | 39.07 | 33.11 | 35.19 | 42.33 |
| 85 | 173.75 | 172.00 | 115.50 | 106.50 | 862.44 | 575.33 | 532.64 | 509.30 | 42.65 | 34.48 | 29.55 | 30.17 |
| 86 | 174.75 | 174.00 | 114.50 | 104.25 | 740.34 | 583.01 | 454.45 | 549.10 | 40.55 | 31.56 | 36.95 | 31.09 |
| 87 | 173.00 | 173.50 | 121.25 | 113.25 | 1053.11 | 726.54 | 740.68 | 625.91 | 38.49 | 28.66 | 38.54 | 38.35 |
| 88 | 172.50 | 174.00 | 122.75 | 115.00 | 881.42 | 734.93 | 561.50 | 563.22 | 45.54 | 34.49 | 28.38 | 30.28 |
| 89 | 174.50 | 174.25 | 120.25 | 123.25 | 871.47 | 606.31 | 589.89 | 569.10 | 41.30 | 35.67 | 35.12 | 33.17 |
| 90 | 175.75 | 176.50 | 122.25 | 119.50 | 1088.15 | 686.67 | 697.53 | 719.68 | 48.61 | 29.73 | 31.91 | 30.96 |
| 91 | 173.00 | 172.75 | 119.75 | 116.50 | 823.62 | 694.16 | 478.55 | 631.92 | 41.74 | 30.72 | 36.81 | 35.83 |
| 92 | 171.50 | 173.00 | 113.75 | 109.75 | 989.56 | 670.98 | 547.62 | 704.57 | 41.88 | 32.80 | 40.36 | 30.91 |
| 93 | 183.00 | 182.50 | 129.00 | 121.00 | 1001.93 | 608.74 | 680.49 | 604.45 | 43.81 | 31.36 | 31.57 | 34.85 |
| 94 | 178.00 | 179.75 | 123.12 | 126.50 | 839.90 | 599.55 | 637.55 | 642.81 | 47.77 | 31.75 | 32.23 | 32.60 |
| 95 | 177.25 | 177.75 | 120.50 | 115.25 | 1013.98 | 739.39 | 779.82 | 850.05 | 42.98 | 24.44 | 28.49 | 36.85 |
| 96 | 176.75 | 177.00 | 114.25 | 111.25 | 1043.75 | 658.97 | 632.14 | 752.35 | 41.17 | 26.60 | 34.69 | 40.69 |
| 97 | 173.00 | 173.50 | 118.37 | 116.75 | 1141.96 | 870.87 | 689.58 | 669.61 | 40.80 | 25.01 | 36.49 | 51.54 |
| 98 | 174.00 | 173.75 | 116.50 | 114.25 | 1176.30 | 887.54 | 711.99 | 709.56 | 40.50 | 29.27 | 41.52 | 45.36 |
| 99 | 174.75 | 174.50 | 125.75 | 118.25 | 910.83 | 673.94 | 692.80 | 696.90 | 44.97 | 32.15 | 31.06 | 30.33 |
| 100 | 178.50 | 174.50 | 113.00 | 117.25 | 1043.94 | 659.23 | 665.79 | 482.03 | 40.07 | 27.33 | 35.55 | 41.51 |
| 102 | 174.50 | 173.75 | 129.00 | 126.50 | 1235.86 | 860.08 | 689.35 | 773.94 | 45.36 | 32.95 | 39.43 | 37.13 |
| 103 | 177.00 | 175.25 | 110.00 | 110.50 | 1072.08 | 698.04 | 734.31 | 655.68 | 44.30 | 29.71 | 37.06 | 35.72 |
| 104 | 178.75 | 176.00 | 117.25 | 116.50 | 1181.75 | 680.81 | 654.54 | 649.49 | 50.58 | 30.72 | 39.19 | 33.65 |
| 105 | 176.50 | 175.50 | 130.50 | 131.25 | 1211.08 | 735.90 | 768.63 | 589.77 | 43.51 | 30.31 | 39.61 | 41.08 |
| 106 | 178.75 | 177.25 | 122.25 | 126.25 | 1086.81 | 693.55 | 831.17 | 728.56 | 39.60 | 27.47 | 36.77 | 32.99 |
| 107 | 176.25 | 177.25 | 127.75 | 131.00 | 1020.17 | 645.29 | 478.64 | 635.69 | 38.02 | 25.88 | 51.19 | 36.23 |
| 108 | 174.25 | 177.75 | 116.75 | 118.00 | 816.70 | 568.52 | 749.00 | 569.29 | 37.89 | 26.62 | 33.00 | 31.47 |
| 109 | 179.00 | 178.25 | 113.75 | 110.25 | 806.30 | 638.59 | 761.17 | 681.09 | 36.14 | 21.62 | 33.53 | 42.79 |
| 110 | 178.00 | 177.25 | 118.62 | 121.00 | 943.59 | 591.64 | 753.69 | 631.02 | 39.36 | 24.99 | 32.68 | 41.76 |
| 111 | 174.50 | 173.50 | 120.50 | 119.75 | 1019.73 | 758.48 | 603.30 | 736.59 | 41.84 | 29.68 | 41.21 | 40.21 |
| 112 | 172.00 | 172.50 | 120.37 | 110.62 | 1018.79 | 551.01 | 804.45 | 601.74 | 44.58 | 35.75 | 27.72 | 31.83 |
| 113 | 174.00 | 173.00 | 120.00 | 112.75 | 1060.26 | 699.88 | 815.70 | 511.86 | 37.70 | 31.98 | 38.70 | 39.10 |
| 114 | 172.50 | 175.75 | 119.00 | 113.37 | 1081.14 | 727.96 | 610.80 | 556.18 | 39.63 | 27.05 | 47.82 | 41.32 |
| 115 | 175.50 | 174.00 | 109.50 | 112.75 | 999.28 | 761.48 | 785.20 | 598.38 | 38.27 | 31.51 | 47.35 | 45.17 |
| 116 | 173.75 | 175.75 | 112.75 | 111.50 | 991.60 | 734.63 | 695.92 | 650.21 | 41.51 | 27.82 | 37.99 | 40.93 |
| 117 | 178.25 | 175.00 | 121.25 | 115.62 | 1099.79 | 666.13 | 719.69 | 579.93 | 40.40 | 28.23 | 37.26 | 40.89 |
| 118 | 173.00 | 175.25 | 115.00 | 116.00 | 1138.20 | 701.50 | 677.74 | 577.27 | 44.10 | 27.30 | 38.19 | 45.46 |
| 119 | 179.25 | 178.25 | 125.25 | 120.87 | 1091.99 | 650.64 | 608.23 | 621.97 | 45.95 | 33.58 | 34.18 | 28.94 |
| 120 | 176.00 | 176.75 | 125.25 | 126.00 | 971.85 | 658.11 | 597.19 | 596.34 | 45.68 | 30.12 | 33.34 | 36.25 |
| 121 | 178.75 | 175.75 | 122.37 | 126.25 | 1063.15 | 739.70 | 610.61 | 649.09 | 48.34 | 31.48 | 34.62 | 36.24 |
| 122 | 175.50 | 175.25 | 124.50 | 124.50 | 1212.99 | 887.28 | 699.71 | 758.94 | 54.00 | 36.68 | 31.55 | 32.72 |
| 123 | 178.25 | 176.75 | 126.62 | 126.12 | 1046.68 | 744.73 | 593.59 | 605.67 | 51.54 | 34.64 | 34.25 | 36.15 |
| 124 | 174.50 | 176.00 | 126.75 | 120.37 | 1221.51 | 802.49 | 743.36 | 696.60 | 39.36 | 27.65 | 44.11 | 49.60 |
| 125 | 178.75 | 174.00 | 122.75 | 116.12 | 974.41 | 567.77 | 629.90 | 471.38 | 39.43 | 31.32 | 35.91 | 43.40 |
| 126 | 176.00 | 175.25 | 111.75 | 113.37 | 1267.36 | 618.01 | 574.14 | 649.31 | 36.73 | 31.26 | 38.95 | 53.22 |
| 127 | 174.50 | 176.50 | 122.75 | 125.12 | 1095.22 | 729.70 | 646.44 | 679.59 | 42.32 | 24.20 | 33.06 | 37.64 |
| 128 | 173.25 | 175.00 | 124.00 | 125.75 | 983.58 | 711.29 | 604.11 | 605.01 | 43.45 | 32.40 | 40.97 | 37.89 |
| 129 | 173.25 | 174.75 | 123.25 | 122.25 | 1015.64 | 664.50 | 704.63 | 518.10 | 43.27 | 35.53 | 36.92 | 38.09 |
| 130 | 175.75 | 178.75 | 131.00 | 128.37 | 1124.05 | 686.99 | 758.43 | 566.65 | 42.55 | 27.53 | 38.24 | 40.69 |
| 131 | 179.50 | 180.25 | 127.62 | 122.37 | 1043.27 | 647.46 | 708.86 | 467.61 | 39.32 | 33.46 | 38.25 | 38.41 |
| 132 | 177.75 | 177.00 | 118.75 | 115.75 | 1020.31 | 659.16 | 666.88 | 660.67 | 40.29 | 25.25 | 39.10 | 38.80 |
| 133 | 181.50 | 176.25 | 122.25 | 119.75 | 1008.64 | 702.88 | 572.66 | 602.03 | 45.64 | 28.43 | 36.30 | 41.76 |
| 135 | 181.75 | 180.75 | 119.25 | 113.50 | 1044.11 | 604.28 | 709.32 | 674.53 | 33.41 | 25.13 | 41.10 | 40.14 |
| 137 | 178.50 | 177.50 | 113.50 | 115.25 | 1066.97 | 577.16 | 747.98 | 770.39 | 37.07 | 23.67 | 35.59 | 34.46 |
| 138 | 177.25 | 175.25 | 117.25 | 117.00 | 1091.18 | 628.80 | 762.92 | 737.54 | 35.70 | 23.88 | 40.40 | 37.47 |
| 139 | 177.75 | 175.00 | 113.00 | 105.87 | 932.94 | 636.21 | 788.36 | 665.17 | 38.30 | 30.22 | 27.56 | 31.57 |
| 141 | 182.50 | 178.50 | 122.75 | 115.37 | 799.61 | 524.26 | 506.72 | 495.54 | 48.72 | 29.20 | 28.27 | 33.28 |
| 142 | 173.00 | 171.75 | 114.75 | 103.37 | 1170.50 | 573.32 | 650.70 | 607.25 | 37.47 | 29.58 | 37.86 | 37.28 |
| 143 | 171.50 | 175.25 | 116.75 | 118.75 | 1217.56 | 883.34 | 728.67 | 699.36 | 43.35 | 32.08 | 36.73 | 42.31 |
| 144 | 172.50 | 174.50 | 116.25 | 123.00 | 956.67 | 698.13 | 588.90 | 755.58 | 38.96 | 24.74 | 39.35 | 38.19 |
| 145 | 179.75 | 178.75 | 120.50 | 122.25 | 1124.26 | 679.02 | 687.15 | 728.25 | 40.34 | 23.36 | 39.58 | 41.27 |
| 146 | 178.25 | 177.75 | 131.00 | 124.75 | 1153.69 | 653.18 | 625.03 | 598.29 | 39.94 | 28.82 | 48.77 | 40.81 |
| 147 | 175.25 | 175.25 | 124.50 | 120.00 | 1184.95 | 695.70 | 641.69 | 501.25 | 43.80 | 31.94 | 41.29 | 40.81 |
| 148 | 176.00 | 177.00 | 132.00 | 120.75 | 1154.75 | 708.29 | 761.08 | 443.80 | 40.55 | 32.78 | 41.31 | 49.52 |
| 149 | 176.50 | 175.75 | 124.87 | 122.00 | 1227.12 | 806.46 | 602.02 | 609.22 | 48.83 | 35.00 | 36.37 | 45.45 |
| 152 | 178.75 | 177.25 | 120.00 | 113.25 | 933.42 | 627.36 | 563.73 | 669.96 | 42.73 | 27.61 | 39.03 | 41.18 |
| 154 | 177.00 | 175.75 | 113.00 | 117.75 | 1193.95 | 779.02 | 835.60 | 810.19 | 38.66 | 24.61 | 36.64 | 35.92 |
| 155 | 177.75 | 175.50 | 117.00 | 112.25 | 1187.01 | 747.92 | 809.47 | 760.56 | 37.50 | 26.01 | 34.67 | 42.22 |
| 156 | 179.50 | 175.50 | 114.62 | 107.00 | 955.62 | 740.12 | 690.94 | 740.90 | 35.91 | 29.66 | 35.41 | 40.31 |
| 157 | 178.50 | 175.25 | 115.62 | 111.75 | 1107.69 | 682.52 | 638.44 | 655.24 | 40.01 | 25.76 | 40.67 | 36.99 |
| 158 | 180.25 | 175.50 | 110.75 | 111.00 | 1050.12 | 686.15 | 803.64 | 692.52 | 42.32 | 26.49 | 26.32 | 39.82 |
| 159 | 172.50 | 172.50 | 118.00 | 116.75 | 1131.04 | 1016.92 | 752.93 | 756.78 | 39.06 | 28.71 | 44.71 | 32.57 |
| 160 | 176.50 | 175.75 | 124.50 | 126.25 | 972.22 | 736.79 | 517.68 | 556.64 | 44.95 | 34.19 | 42.32 | 43.49 |
| 161 | 176.50 | 174.25 | 120.00 | 118.25 | 1072.32 | 827.55 | 586.71 | 644.53 | 41.50 | 28.73 | 44.84 | 37.22 |
| 165 | 179.25 | 175.75 | 123.50 | 124.50 | 915.29 | 795.18 | 719.74 | 891.52 | 38.39 | 24.35 | 37.30 | 43.99 |
| 166 | 176.00 | 174.00 | 119.75 | 117.25 | 940.13 | 592.31 | 420.42 | 518.54 | 42.19 | 25.70 | 42.94 | 35.41 |
| 167 | 170.50 | 172.75 | 108.50 | 115.50 | 1027.49 | 638.36 | 626.85 | 540.49 | 36.41 | 24.68 | 45.10 | 40.09 |
| 168 | 178.00 | 176.00 | 111.25 | 111.25 | 1133.30 | 786.40 | 818.26 | 745.21 | 34.49 | 27.24 | 40.76 | 47.19 |
| 169 | 177.75 | 176.25 | 113.75 | 113.75 | 1142.66 | 671.61 | 652.46 | 616.26 | 41.97 | 25.51 | 39.51 | 44.33 |
| 170 | 178.75 | 178.00 | 113.25 | 115.75 | 980.83 | 610.91 | 603.59 | 541.35 | 39.30 | 25.07 | 40.19 | 44.78 |
| 172 | 178.00 | 175.50 | 115.00 | 119.25 | 971.60 | 630.03 | 751.42 | 732.46 | 41.23 | 22.59 | 32.98 | 45.92 |
| 173 | 177.50 | 175.25 | 118.50 | 112.62 | 903.61 | 643.24 | 767.30 | 793.92 | 37.70 | 25.92 | 30.53 | 34.22 |
| 174 | 179.00 | 177.50 | 112.25 | 111.25 | 1055.39 | 615.90 | 682.48 | 594.46 | 46.79 | 28.17 | 35.42 | 32.41 |
| 175 | 178.75 | 176.75 | 114.75 | 113.25 | 954.53 | 621.79 | 602.16 | 645.34 | 44.46 | 28.22 | 32.90 | 38.00 |
| 176 | 179.50 | 176.25 | 122.62 | 113.25 | 1054.58 | 728.05 | 791.22 | 704.17 | 36.36 | 23.90 | 35.21 | 35.99 |
| 177 | 175.25 | 177.00 | 113.00 | 109.75 | 962.06 | 645.12 | 679.31 | 564.78 | 37.49 | 26.49 | 40.95 | 35.34 |
| 178 | 175.25 | 176.75 | 115.75 | 114.50 | 889.57 | 653.02 | 694.78 | 583.51 | 38.06 | 25.02 | 38.13 | 46.22 |
| 179 | 175.00 | 175.00 | 118.50 | 116.87 | 1050.91 | 673.17 | 805.27 | 605.03 | 35.73 | 26.24 | 39.54 | 42.40 |
| 180 | 174.00 | 177.50 | 112.62 | 112.00 | 980.82 | 631.22 | 691.34 | 618.52 | 44.59 | 23.27 | 35.49 | 42.52 |
| 181 | 175.00 | 174.00 | 114.25 | 110.50 | 1010.73 | 717.13 | 701.94 | 584.54 | 40.37 | 28.27 | 40.38 | 36.79 |
| 182 | 173.50 | 175.75 | 112.75 | 110.12 | 977.71 | 596.51 | 661.78 | 596.27 | 43.25 | 31.09 | 37.99 | 43.13 |
| 183 | 177.50 | 175.25 | 112.25 | 107.75 | 1036.52 | 736.63 | 614.58 | 589.43 | 43.80 | 30.27 | 44.29 | 30.64 |
| 184 | 176.25 | 176.75 | 114.25 | 115.50 | 1051.41 | 624.80 | 702.44 | 509.49 | 41.73 | 28.48 | 37.60 | 43.58 |
| 185 | 178.75 | 177.25 | 115.75 | 110.50 | 908.79 | 646.94 | 750.51 | 697.77 | 38.06 | 23.12 | 33.40 | 41.05 |
| 186 | 177.50 | 176.25 | 118.25 | 113.25 | 1068.11 | 680.97 | 612.14 | 526.47 | 47.79 | 32.36 | 37.66 | 37.72 |
| 187 | 176.75 | 176.00 | 118.00 | 118.25 | 902.49 | 813.88 | 646.46 | 926.20 | 42.45 | 23.16 | 35.69 | 37.58 |
| 188 | 174.25 | 173.25 | 111.62 | 111.50 | 947.32 | 643.98 | 485.76 | 399.20 | 40.74 | 27.85 | 42.52 | 35.98 |
| 189 | 173.25 | 176.50 | 112.00 | 109.25 | 808.16 | 675.83 | 742.49 | 717.23 | 38.16 | 26.02 | 30.24 | 45.25 |
| 190 | 178.50 | 173.75 | 117.25 | 102.75 | 852.86 | 829.57 | 779.73 | 757.16 | 37.26 | 27.26 | 29.45 | 30.34 |
| 191 | 176.00 | 177.25 | 115.25 | 105.25 | 1053.46 | 662.20 | 898.58 | 676.79 | 39.47 | 25.94 | 31.10 | 33.19 |
| 192 | 176.75 | 175.75 | 114.50 | 111.75 | 816.80 | 724.18 | 715.99 | 628.50 | 40.74 | 29.55 | 27.45 | 36.59 |
| 193 | 176.25 | 175.25 | 115.00 | 113.00 | 1028.62 | 690.16 | 880.48 | 767.88 | 41.02 | 26.05 | 33.59 | 36.92 |
| 196 | 175.50 | 173.75 | 115.25 | 112.50 | 1056.35 | 938.97 | 706.73 | 626.77 | 38.89 | 28.49 | 45.22 | 32.63 |
| 197 | 174.25 | 175.50 | 107.50 | 110.37 | 916.98 | 729.24 | 742.43 | 653.02 | 34.44 | 25.40 | 36.08 | 50.16 |
| 198 | 176.25 | 175.00 | 108.75 | 108.37 | 1085.43 | 889.85 | 839.68 | 650.74 | 33.48 | 31.15 | 42.98 | 40.74 |
| 199 | 174.75 | 174.50 | 111.00 | 108.75 | 1008.31 | 926.71 | 718.55 | 710.94 | 39.13 | 28.07 | 40.31 | 42.22 |
| 200 | 175.25 | 176.50 | 114.00 | 111.50 | 865.18 | 805.54 | 664.15 | 645.97 | 36.39 | 25.90 | 42.04 | 44.94 |
| 201 | 175.75 | 176.25 | 107.50 | 113.25 | 1063.72 | 799.48 | 801.43 | 741.61 | 37.17 | 25.95 | 35.67 | 41.86 |
| 202 | 174.50 | 173.50 | 114.00 | 108.75 | 849.02 | 894.65 | 674.85 | 685.76 | 42.49 | 32.73 | 34.77 | 37.55 |
| 203 | 176.00 | 175.00 | 120.75 | 116.25 | 859.04 | 662.97 | 596.66 | 479.32 | 41.16 | 33.15 | 36.09 | 42.00 |
| 204 | 176.75 | 174.25 | 116.50 | 114.37 | 1088.68 | 901.57 | 801.49 | 654.63 | 48.14 | 37.18 | 30.07 | 29.20 |
| 205 | 177.00 | 176.75 | 121.37 | 119.75 | 842.41 | 494.47 | 420.65 | 455.82 | 42.39 | 20.69 | 40.55 | 40.09 |
| 206 | 179.75 | 181.25 | 135.50 | 143.50 | 776.39 | 601.48 | 605.63 | 527.69 | 43.74 | 29.10 | 38.23 | 35.50 |
| 207 | 180.50 | 175.25 | 114.75 | 103.75 | 721.52 | 500.22 | 483.98 | 445.46 | 29.78 | 21.73 | 29.38 | 40.83 |
| 208 | 179.25 | 177.50 | 117.00 | 127.00 | 895.13 | 741.85 | 636.46 | 664.41 | 31.05 | 21.30 | 45.88 | 48.15 |
| LSD | 3.70 | 2.68 | 8.80 | 8.50 | 241.56 | 212.18 | 226.66 | 201.27 | 6.48 | 5.64 | 7.74 | 7.50 |

| **Table S3.** Population of 184 synthetic derived hexaploid wheat lines from CIMMYT. Genotype identification (GID), introduction number (INTRID), Cross identification (CID), Synthetic Identification (SID), pedigree and synthetic degree are provided. The diploid D-genome donor (*Aegilops tauschii*) accession used in the synthetic cross is bolded and the tetraploid AB genome donor accession (*Triticum turgidum*) used in the cross is underlined. | | | | | | |
| --- | --- | --- | --- | --- | --- | --- |
|  | | | | | | |
| **Genotype code** | **GID** | **INTRID** | **CID** | **SID** | **Synthetic degree** | **Pedigree** |
| **Synthetic wheat** | |  |  |  |  |  |
| 7 | 2454874 | BW31682 | 72683 | 805 | 2^nd^ | Altar84/***Ae.Squarrosa*(219)**//SeriM 82 |
| 8 | 2454873 | BW31683 | 72683 | 806 | 2^nd^ | Altar84/***Ae.Squarrosa*(219)**//SeriM 82 |
| 9 | 2454876 | BW31684 | 72726 | 648 | 2^nd^ | Croc_1/***Ae.Squarrosa*(224)**//OpataM 85 |
| 10 | 1118879 | BW31690 | 72726 | 533 | 2^nd^ | Croc_1/***Ae.Squarrosa*(224)**//OpataM 85 |
| 11 | 2454972 | BW31691 | 72744 | 375 | 2^nd^ | Dverd_2/***Ae.Squarrosa*(214)**//OpataM85 |
| 12 | 2454971 | BW31692 | 72744 | 376 | 2^nd^ | Dverd_2/***Ae.Squarrosa*(214)**//OpataM85 |
| 13 | 2454970 | BW31693 | 72744 | 377 | 2^nd^ | Dverd_2/***Ae.Squarrosa*(214)**//OpataM85 |
| 14 | 2454969 | BW31694 | 72744 | 378 | 2^nd^ | Dverd_2/***Ae.Squarrosa*(214)**//OpataM85 |
| 15 | 2454968 | BW31695 | 72744 | 379 | 2^nd^ | Dverd_2/***Ae.Squarrosa*(214)**//OpataM85 |
| 16 | 2454967 | BW31696 | 72744 | 380 | 2^nd^ | Dverd_2/***Ae.Squarrosa*(214)**//OpataM85 |
| 17 | 2454966 | BW31697 | 72744 | 381 | 2^nd^ | Dverd_2/***Ae.Squarrosa*(214)**//OpataM85 |
| 18 | 1403554 | BW31725 | 101714 | 73 | 3^rd^ | Croc_1/***Ae.Squarrosa*(205)**//JupatecoF 73/Bluejay/3/Super Kauz/4/Kauz |
| 19 | 2479188 | BW31780 | 167256 | 307 | 2^nd^ | Bacanora T88//Sora/***Ae.Squarrosa*** **(323)** |
| 20 | 2479187 | BW31781 | 167256 | 308 | 2^nd^ | Bacanora T88//Sora/***Ae.Squarrosa*** **(323)** |
| 21 | 2479186 | BW31782 | 167256 | 309 | 2^nd^ | Bacanora T88//Sora/***Ae.Squarrosa*** **(323)** |
| 22 | 2479185 | BW31783 | 167256 | 310 | 2^nd^ | Bacanora T88//Sora/***Ae.Squarrosa*** **(323)** |
| 23 | 1874471 | BW31784 | 167282 | 572 | 2^nd^ | Bacanora T88//Croc_1/***Ae.Squarrosa*** **(662)** |
| 24 | 1874485 | BW31785 | 167282 | 567 | 2^nd^ | Bacanora T88//Croc_1/***Ae.Squarrosa*** **(662)** |
| 25 | 1874482 | BW31786 | 167282 | 568 | 2^nd^ | Bacanora T88//Croc_1/***Ae.Squarrosa*** **(662)** |
| 26 | 1874480 | BW31787 | 167282 | 569 | 2^nd^ | Bacanora T88//Croc_1/***Ae.Squarrosa*** **(662)** |
| 27 | 1874477 | BW31788 | 167282 | 570 | 2^nd^ | Bacanora T88//Croc_1/***Ae.Squarrosa*** **(662)** |
| 28 | 1874474 | BW31789 | 167282 | 571 | 2^nd^ | Bacanora T88//Croc_1/***Ae.Squarrosa*** **(662)** |
| 29 | 1874460 | BW31790 | 167282 | 577 | 2^nd^ | Bacanora T88//Croc_1/***Ae.Squarrosa*** **(662)** |
| 30 | 1874466 | BW31791 | 167282 | 574 | 2^nd^ | Bacanora T88//Croc_1/***Ae.Squarrosa*** **(662)** |
| 31 | 1874462 | BW31792 | 167282 | 576 | 2^nd^ | Bacanora T88//Croc_1/***Ae.Squarrosa*** **(662)** |
| 32 | 1874444 | BW31793 | 167282 | 583 | 2^nd^ | Bacanora T88//Croc_1/***Ae.Squarrosa*** **(662)** |
| 33 | 1874454 | BW31794 | 167282 | 579 | 2^nd^ | Bacanora T88//Croc_1/***Ae.Squarrosa*** **(662)** |
| 34 | 1874446 | BW31796 | 167282 | 582 | 2^nd^ | Bacanora T88//Croc_1/***Ae.Squarrosa*** **(662)** |
| 35 | 1874426 | BW31797 | 167282 | 590 | 2^nd^ | Bacanora T88//Croc_1/***Ae.Squarrosa*** **(662)** |
| 36 | 1874431 | BW31798 | 167282 | 588 | 2^nd^ | Bacanora T88//Croc_1/***Ae.Squarrosa*** **(662)** |
| 37 | 1874392 | BW31799 | 167282 | 603 | 2^nd^ | Bacanora T88//Croc_1/***Ae.Squarrosa*** **(662)** |
| 38 | 1874387 | BW31801 | 167282 | 605 | 2^nd^ | Bacanora T88//Croc_1/***Ae.Squarrosa*** **(662)** |
| 39 | 2479415 | BW31802 | 167290 | 197 | 2^nd^ | Bacanora T88//Yarmuk/***Ae.Squarrosa*** **(434)** |
| 40 | 2479414 | BW31803 | 167290 | 198 | 2^nd^ | Bacanora T88//Yarmuk/***Ae.Squarrosa*** **(434)** |
| 41 | 2479413 | BW31804 | 167290 | 199 | 2^nd^ | Bacanora T88//Yarmuk/***Ae.Squarrosa*** **(434)** |
| 42 | 2479417 | BW31805 | 167295 | 347 | 2^nd^ | Bacanora T88/4/Rabi/Ganso/Crane/3/ ***Ae.Squarrosa*** **(895)** |
| 43 | 1877008 | BW31806 | 167357 | 95 | 2^nd^ | Opata M85//Decoy 1/***Ae.Squarrosa*** **(510)** |
| 44 | 1877014 | BW31807 | 167357 | 93 | 2^nd^ | Opata M85//Decoy 1/***Ae.Squarrosa*** **(510)** |
| 45 | 1877011 | BW31808 | 167357 | 94 | 2^nd^ | Opata M85//Decoy 1/***Ae.Squarrosa*** **(510)** |
| 46 | 1877995 | BW31810 | 167384 | 165 | 2^nd^ | Opata M85//Croc 1/***Ae.Squarrosa*** **(879)** |
| 47 | 1877998 | BW31811 | 167384 | 164 | 2^nd^ | Opata M85//Croc 1/***Ae.Squarrosa*** **(879)** |
| 48 | 1877986 | BW31812 | 167384 | 168 | 2^nd^ | Opata M85//Croc 1/***Ae.Squarrosa*** **(879)** |
| 49 | 1877980 | BW31814 | 167384 | 170 | 2^nd^ | Opata M85//Croc 1/***Ae.Squarrosa*** **(879)** |
| 50 | 1118891 | BW32103 | 72726 | 530 | 2^nd^ | Croc 1/***Ae.Squarrosa*** **(224)**//Opata M85 |
| 51 | 1118885 | BW32105 | 72726 | 532 | 2^nd^ | Croc 1/***Ae.Squarrosa*** **(224)**//Opata M85 |
| 52 | 2454893 | BW32106 | 72726 | 630 | 2^nd^ | Croc 1/***Ae.Squarrosa*** **(224)**//Opata M85 |
| 53 | 2454892 | BW32107 | 72726 | 631 | 2^nd^ | Croc 1/***Ae.Squarrosa*** **(224)**//Opata M85 |
| 54 | 1403557 | BW32114 | 101714 | 72 | 4^th^ | Croc 1/***Ae.Squarrosa*** **(205)**//Jupateco F73/Bluejay/3/Super Kauz/4/K |
| 55 | 2453300 | BW32132 | 58520 | 34 | 2^nd^ | Chen/***Ae.Squarrosa***//2*Opata M85 |
| 56 | 2453315 | BW32133 | 58561 | 34 | 2^nd^ | Altar 84/***Ae.Squarrosa***//2*Opata M85 |
| 57 | 346421 | BW33624 | 54005 | 193 | 2^nd^ | Altar 84/***Aegilops Squarrosa(Taus)***//2*Opata M85 |
| 58 | 1118888 | BW33631 | 72726 | 531 | 2^nd^ | Croc 1/***Ae.Squarrosa*** **(224)**//Opata M85 |
| 59 | 2478016 | BW33659 | 158324 | 50 | 3^rd^ | Pastor//Sitella/Mochis73/3/Chen/***Aegilops Squarrosa*** **(*Taus*)**//Bacanora T88 |
| 60 | 2478015 | BW33660 | 158324 | 51 | 3^rd^ | Pastor//Sitella/Mochis73/3/Chen/***Aegilops Squarrosa*** **(*Taus*)**//Bacanora T88 |
| 61 | 2478024 | BW33661 | 158324 | 42 | 3^rd^ | Pastor//Sitella/Mochis73/3/Chen/***Aegilops Squarrosa*** **(*Taus*)**//Bacanora T88 |
| 62 | 2478023 | BW33662 | 158324 | 43 | 3^rd^ | Pastor//Sitella/Mochis73/3/Chen/***Aegilops Squarrosa*** **(*Taus*)**//Bacanora T88 |
| 63 | 2478033 | BW33665 | 158325 | 44 | 3^rd^ | Pastor/3/Munia//Chen/Altar 84/5/Cando/R143//Ente/Mexicali 2/3/***Aegilops Squarrosa*** **(*Taus*)**/4/Weaver |
| 64 | 3616958 | BW33666 | 158452 | 135 | 3^rd^ | Filin/Irena/5/Cando/R143//Ente/Mexicali 2/3/***Aegilops Squarrosa*** **(*Taus*)**/4/Weaver |
| 65 | 3616956 | BW33667 | 158452 | 132 | 3^rd^ | Filin/Irena/5/Cando/R143//Ente/Mexicali 2/3/***Aegilops Squarrosa*** **(*Taus*)**/4/Weaver |
| 66 | 3567694 | BW33670 | 158452 | 131 | 3^rd^ | Filin/Irena/5/Cando/R143//Ente/Mexicali 2/3/***Aegilops Squarrosa*** **(*Taus*)**/4/Weaver |
| 67 | 3632351 | BW33680 | 255660 | 97 | 3^rd^ | Chbia/5/Cando/R143//Ente/Mexicali 2/3/***Aegilops Squarrosa*** **(*Taus*)**/4/Weaver |
| 68 | 3577944 | BW33685 | 206381 | 227 | 3^rd^ | Croc 1/***Ae.Squarrosa*** **(213)**//Papago M86/3/CMH81.38/2*Kauz |
| 69 | 3577945 | BW33686 | 206381 | 225 | 3^rd^ | Croc 1/***Ae.Squarrosa*** **(213)**//Papago M86/3/CMH81.38/2*Kauz |
| 70 | 3628179 | BW33687 | 206381 | 226 | 3^rd^ | Croc 1/***Ae.Squarrosa*** **(213)**//Papago M86/3/CMH81.38/2*Kauz |
| 71 | 3628190 | BW33688 | 206499 | 26 | 2^nd^ | Altar 84/***Ae.Squarrosa*** **(219)**//Attila |
| 72 | 3567695 | BW33689 | 207697 | 27 | 3^rd^ | Cando/R143//Ente/Mexicali 2/3/***Aegilops Squarrosa*** **(*Taus*)**/4/Ocoroni F86/5/Pastor |
| 73 | 3628930 | BW33691 | 207963 | 31 | 3^rd^ | Munia/3/Ruff/Flamingo Dr//Yavaros79/4/Chen/***Aegilops Squarrosa (Taus)***//Bacanora T 88 |
| 74 | 3628949 | BW33693 | 207970 | 130 | 3^rd^ | Hoopoe/Tanager//Veery/3/2*Papago M86/4/Chen/***Aegilops Squarrosa (Taus)***//Bacanora T 88 |
| 76 | 3628971 | BW33695 | 207990 | 99 | 3^rd^ | Fasan/2*Tepoca T89/3/Chen/***Aegilops Squarrosa (Taus)***//Bacanora T 88 |
| 77 | 3631236 | BW33728 | 210539 | 129 | 3^rd^ | Croc 1/***Ae.Squarrosa*** **(224)**//Opata M85/Kauz*2/Bobwhite//Kauz/4/NL 683 |
| 78 | 3631235 | BW33729 | 210539 | 130 | 3^rd^ | Croc 1/***Ae.Squarrosa*** **(224)**//Opata M85/Kauz*2/Bobwhite//Kauz/4/NL 683 |
| 79 | 3631234 | BW33731 | 210539 | 131 | 3^rd^ | Croc 1/***Ae.Squarrosa*** **(224)**//Opata M85/Kauz*2/Bobwhite//Kauz/4/NL 683 |
| 80 | 3621025 | BW33746 | 167276 | 215 | 2^nd^ | Bacanora T88//Cerceta/***Ae.Searsii*** **(34D)** |
| 81 | 3621024 | BW33747 | 167276 | 216 | 2^nd^ | Bacanora T88//Cerceta/***Ae.Searsii*** **(34D)** |
| 82 | 3616097 | BW33757 | 152383 | 95 | 3^rd^ | Altar 84/***Ae.Squarrosa* (224)**/Cucurpe S86/3/PI 610755 |
| 83 | 2479441 | BW33758 | 167370 | 255 | 2^nd^ | Opata M85//Sora/***Ae.Squarrosa*** **(323)** |
| 84 | 1799716 | BW33904 | 158634 | 243 | 3^rd^ | Munia/Chorlito/Pfau/Bobwhite//Veery #9/4/Chen/***Aegilops Squarrosa (Taus)***//Bacanora T 88 |
| 85 | 3584454 | BW33952 | 135084 | 149 | 3^rd^ | Croc 1/***Ae.Squarrosa*** **(205)**//Borlaug M95/3/2*Milan |
| 86 | 2478018 | BW33977 | 158324 | 48 | 3^rd^ | Pastor//Sitella/Mochis73/3/Chen/***Aegilops Squarrosa*** **(*Taus*)**//Bacanora T88 |
| 87 | 3567684 | BW33979 | 206280 | 223 | 3^rd^ | Croc 1/***Ae.Squarrosa*** **(205)**//Kauz/3/Eneida F94 |
| 88 | 2478027 | BW35684 | 158325 | 50 | 3^rd^ | Pastor/3/Munia//Chen/Altar 84/5/Cando/R143//Ente/Mexicali_2/3/***Aegilops Squarrosa*** **(*Taus*)**/4/Weaver |
| 89 | 3855011 | BW35697 | 279807 | 61 | 3^rd^ | Croc 1/***Ae.Squarrosa*** **(224)**//Opata M85/3/Pastor |
| 90 | 3827768 | BW35698 | 279807 | 53 | 3^rd^ | Croc 1/***Ae.Squarrosa*** **(224)**//Opata M85/3/Pastor |
| 91 | 3605425 | BW36364 | 118849 | 366 | 3^rd^ | Croc 1/***Ae.Squarrosa*** **(205)**//Kauz/3/Sasia |
| 92 | 2478022 | BW36375 | 158324 | 44 | 3^rd^ | Pastor//Sitella/Mochis73/3/Chen/***Aegilops Squarrosa*** **(*Taus*)**//Bacanora T88 |
| 93 | 3574436 | BW36377 | 158452 | 133 | 3^rd^ | Filin/Irena/5/Cando/R143//Ente/Mexicali 2/3/***Aegilops Squarrosa*** **(*Taus*)**/4/Weaver |
| 94 | 3616959 | BW36378 | 158452 | 134 | 3^rd^ | Filin/Irena/5/Cando/R143//Ente/Mexicali 2/3/***Aegilops Squarrosa*** **(*Taus*)**/4/Weaver |
| 95 | 3586075 | BW36379 | 255660 | 102 | 3^rd^ | Chibia/5/Cando/R143//Ente/Mexicali 2/3/***Aegilops Squarrosa*** **(*Taus*)**/4/Weaver |
| 96 | 3574425 | BW36380 | 255660 | 100 | 3^rd^ | Chibia/5/Cando/R143//Ente/Mexicali 2/3/***Aegilops Squarrosa*** **(*Taus*)**/4/Weaver |
| 97 | 3827946 | BW36535 | 304079 | 106 | 3^rd^ | Caskor/3/Croc_1/***Ae. Squarrosa*(224)**//Opata M85 |
| 98 | 3864981 | BW36536 | 304079 | 109 | 3^rd^ | Caskor/3/Croc_1/***Ae. Squarrosa*(224)**//Opata M85 |
| 99 | 3827947 | BW36542 | 279807 | 56 | 3^rd^ | Croc_1/***Ae. Squarrosa*(224)**//Opata M85/3/Pastor |
| 100 | 3855644 | BW36550 | 280725 | 76 | 3^rd^ | Pajonal/Bobwhite//Opata M85*2/3/Croc_1/***Ae. Squarrosa*(224)**//Opata M85 |
| 102 | 3855902 | BW36555 | 280805 | 59 | 4^th^ | Milan/Kauz/5/Cando/R143//Ente/Mexicali_2/3/***Aegilops Squarrosa*** **(*Taus*)**/4/Weaver/6/Tobari F66/Era//Tobari F66/Ciano F67/3/Pollo/4/Veery#5/5/Kauz |
| 103 | 3827938 | BW36560 | 334948 | 269 | 3^rd^ | Pastor/3/Altar 84/***Aegilops Squarrosa*** **(*Taus*)**//Opata M85 |
| 104 | 3827751 | BW36561 | 334948 | 272 | 3^rd^ | Pastor/3/Altar 84/***Aegilops Squarrosa*** **(*Taus*)**//Opata M85 |
| 105 | 3888537 | BW36562 | 334948 | 301 | 3^rd^ | Pastor/3/Altar 84/***Aegilops Squarrosa*** **(*Taus*)**//Opata M85 |
| 106 | 3888335 | BW36563 | 334948 | 307 | 3^rd^ | Pastor/3/Altar 84/***Aegilops Squarrosa*** **(*Taus*)**//Opata M85 |
| 107 | 3888320 | BW36564 | 334948 | 313 | 3^rd^ | Pastor/3/Altar 84/***Aegilops Squarrosa*** **(*Taus*)**//Opata M85 |
| 108 | 4314513 | BW36566 | 335446 | 55 | 3^rd^ | Croc_1/***Ae. Squarrosa*(213)**//Papago M86/3/Baviacora M92 |
| 109 | 4315350 | BW36568 | 331793 | 57 | 3^rd^ | Altar 84/***Ae.Squarrosa*** **(221)**//Pastor/3/Pastor |
| 110 | 2489102 | BW36874 | 255660 | 81 | 3^rd^ | Chibia/5/Cando/R143//Ente/Mexicali 2/3/***Aegilops Squarrosa*** **(*Taus*)**/4/Weaver |
| 111 | 2489138 | BW36875 | 255700 | 32 | 3^rd^ | Capeiti 8/5/Gediz 73/3/Goose//Albatros:Dr/Crane/4/***Ae. Squarrosa*** **(208)**/5/Hahn/2*Weaver |
| 112 | 2489133 | BW36876 | 255700 | 37 | 3^rd^ | Capeiti 8/5/Gediz 73/3/Goose//Albatros:Dr/Crane/4/***Ae. Squarrosa*** **(208)**/5/Hahn/2*Weaver |
| 113 | 3864983 | BW36878 | 304079 | 107 | 3^rd^ | Caskor/3/Croc_1/***Ae. Squarrosa*(224)**//Opata M85 |
| 114 | 3829903 | BW36879 | 304079 | 110 | 3^rd^ | Caskor/3/Croc_1/***Ae. Squarrosa*(224)**//Opata M85 |
| 115 | 3865440 | BW36880 | 304415 | 20 | 2^nd^ | Croc 1/***Ae.Squarrosa*** **(205)**//Milan//Kauz |
| 116 | 3865439 | BW36881 | 304415 | 21 | 2^nd^ | Croc 1/***Ae.Squarrosa*** **(205)**//Milan//Kauz |
| 117 | 3865438 | BW36882 | 304415 | 22 | 2^nd^ | Croc 1/***Ae.Squarrosa*** **(205)**//Milan//Kauz |
| 118 | 3865437 | BW36883 | 304415 | 23 | 2^nd^ | Croc 1/***Ae.Squarrosa*** **(205)**//Milan//Kauz |
| 119 | 3855024 | BW36898 | 279807 | 55 | 3^rd^ | Croc 1/***Ae.Squarrosa*** **(224)**//Opata M85/3/Pastor |
| 120 | 3855017 | BW36900 | 279807 | 64 | 3^rd^ | Croc 1/***Ae.Squarrosa*** **(224)**//Opata M85/3/Pastor |
| 121 | 3855014 | BW36901 | 279807 | 67 | 3^rd^ | Croc 1/***Ae.Squarrosa*** **(224)**//Opata M85/3/Pastor |
| 122 | 3855013 | BW36902 | 279807 | 68 | 3^rd^ | Croc 1/***Ae.Squarrosa*** **(224)**//Opata M85/3/Pastor |
| 123 | 3855012 | BW36903 | 279807 | 69 | 3^rd^ | Croc 1/***Ae.Squarrosa*** **(224)**//Opata M85/3/Pastor |
| 124 | 3855034 | BW36904 | 279810 | 59 | 3^rd^ | Croc 1/***Ae.Squarrosa*** **(224)**//Opata M85/3/Pastor |
| 125 | 3855656 | BW36921 | 280725 | 64 | 3^rd^ | Pajonal/Bobwhite//Opata M85*2/3/Croc 1/***Ae.Squarrosa*** **(224)**//Opata M85 |
| 126 | 3855652 | BW36922 | 280725 | 68 | 3^rd^ | Pajonal/Bobwhite//Opata M85*2/3/Croc 1/***Ae.Squarrosa*** **(224)**//Opata M85 |
| 127 | 3855647 | BW36923 | 280725 | 73 | 3^rd^ | Pajonal/Bobwhite//Opata M85*2/3/Croc 1/***Ae.Squarrosa*** **(224)**//Opata M85 |
| 128 | 3855900 | BW36935 | 280805 | 61 | 4^th^ | Milan/Kauz/5/Cando/R143//Ente/Mexicali_2/3/***Aegilops Squarrosa*** **(*Taus*)**/4/Weaver/6/Tobari F66/Era//Tobari F66/Ciano F67/3/Pollo/4/Veery#5/5/Kauz |
| 129 | 3855898 | BW36937 | 280805 | 64 | 4^th^ | Milan/Kauz/5/Cando/R143//Ente/Mexicali_2/3/***Aegilops Squarrosa*** **(*Taus*)**/4/Weaver/6/Tobari F66/Era//Tobari F66/Ciano F67/3/Pollo/4/Veery#5/5/Kauz |
| 130 | 3888364 | BW36951 | 334948 | 280 | 3^rd^ | Pastor/3/Altar 84/***Aegilops Squarrosa*** **(*Taus*)**//Opata M85 |
| 131 | 3888342 | BW36952 | 334948 | 287 | 3^rd^ | Pastor/3/Altar 84/***Aegilops Squarrosa*** **(*Taus*)**//Opata M85 |
| 132 | 3888359 | BW36953 | 334948 | 294 | 3^rd^ | Pastor/3/Altar 84/***Aegilops Squarrosa*** **(*Taus*)**//Opata M85 |
| 133 | 3888358 | BW36954 | 334948 | 295 | 3^rd^ | Pastor/3/Altar 84/***Aegilops Squarrosa*** **(*Taus*)**//Opata M85 |
| 135 | 3888349 | BW36956 | 334948 | 264 | 3^rd^ | Pastor/3/Altar 84/***Aegilops Squarrosa*** **(*Taus*)**//Opata M85 |
| 137 | 4340793 | BW36965 | 331793 | 56 | 3^rd^ | Altar 84/***Ae. Squarrosa*** **(221)**//Pastor/3/Pastor |
| 138 | 4352598 | BW36979 | 341652 | 48 | 3^rd^ | Altar 84/***Ae. Squarrosa*** **(193)**//2*Pastor |
| 139 | 2489095 | BW36981 | 255660 | 88 | 3^rd^ | Chibia/5/Cando/R143//Ente/Mexicali 2/3/***Aegilops Squarrosa*** **(*Taus*)**/4/Weaver |
| 141 | 3888399 | BW36984 | 334948 | 517 | 3^rd^ | Pastor/3/Altar 84/***Aegilops Squarrosa*** **(*Taus*)**//Opata M85 |
| 142 | 3833280 | BW37454 | 72726 | 797 | 2^nd^ | Croc 1/***Ae.Squarrosa*** **(224)**//Opata M85 |
| 143 | 4563458 | BW37461 | 303992 | 57 | 3^rd^ | Cando/R143//Ente/Mexicali 2/3/***Aegilops Squarrosa*** **(*Taus*)**/4/Weaver/5/Pastor |
| 144 | 4093487 | BW37467 | 304079 | 120 | 3^rd^ | Caskor/3/Croc_1/***Ae. Squarrosa*(224)**//Opata M85 |
| 145 | 4569087 | BW37505 | 334948 | 1076 | 3^rd^ | Pastor/3/Altar 84/***Aegilops Squarrosa*** **(*Taus*)**//Opata M85 |
| 146 | 4569081 | BW37507 | 334948 | 1092 | 3^rd^ | Pastor/3/Altar 84/***Aegilops Squarrosa*** **(*Taus*)**//Opata M85 |
| 147 | 4569074 | BW37508 | 334948 | 1099 | 3^rd^ | Pastor/3/Altar 84/***Aegilops Squarrosa*** **(*Taus*)**//Opata M85 |
| 148 | 4569073 | BW37509 | 334948 | 1100 | 3^rd^ | Pastor/3/Altar 84/***Aegilops Squarrosa*** **(*Taus*)**//Opata M85 |
| 149 | 4577962 | BW37534 | 342469 | 53 | 3^rd^ | Milan/Kauz/3/Ures T81/Junco/Kauz/4/Croc 1/***Ae.Squarrosa*** **(224)**//Opata M85 |
| 152 | 4773725 | BW37606 | 365653 | 51 | 3^rd^ | Altar 84/***Aegilops Squarrosa*** **(*Taus*)**//Ocoroni F86/3/Veery/Marcos Juarez Inta/2*Tui |
| 154 | 3832784 | BW37608 | 58520 | 92 | 2^nd^ | Chen/***Ae.Squarrosa***//2*Opata M85 |
| 155 | 3832819 | BW37609 | 58561 | 111 | 3^rd^ | Altar 84/***Ae.Squarrosa***//2*Opata M85 |
| 156 | 3832827 | BW37610 | 58561 | 103 | 3^rd^ | Altar 84/***Ae.Squarrosa***//2*Opata M85 |
| 157 | 4563455 | BW37683 | 303992 | 60 | 3^rd^ | Cando/R143//Ente/Mexicali 2/3/***Aegilops Squarrosa*** **(*Taus*)**/4/Weaver/5/Pastor |
| 158 | 4062607 | BW37698 | 334948 | 1102 | 3^rd^ | Pastor/3/Altar 84/***Aegilops Squarrosa*** **(*Taus*)**//Opata M852 |
| 159 | 4577785 | BW37701 | 342438 | 56 | 3^rd^ | SuperKauz/Pastor/3/Croc_1/***Ae. Squarrosa*(224)**//Opata M85 |
| 160 | 4577963 | BW37706 | 342469 | 52 | 3^rd^ | Milan/Kauz/3/Ures T81/Junco/Kauz/4/Croc 1/***Ae.Squarrosa*** **(224)**//Opata M85 |
| 161 | 4753157 | BW37707 | 342488 | 59 | 3^rd^ | Kaby/Baviacora M92/3/Croc 1/***Ae.Squarrosa*** **(224)**//Opata M85 |
| 165 | 4342466 | BW37856 | 334948 | 1086 | 3^rd^ | Pastor/3/Altar 84/***Aegilops Squarrosa*** **(*Taus*)**//Opata M852 |
| 166 | 4569078 | BW37857 | 334948 | 1095 | 3^rd^ | Pastor/3/Altar 84/***Aegilops Squarrosa*** **(*Taus*)**//Opata M852 |
| 167 | 4577760 | BW37864 | 342432 | 61 | 3^rd^ | SuperKauz/Baviacora M92/3/Croc_1/***Ae. Squarrosa*(224)**//Opata M85 |
| 168 | 4753160 | BW37867 | 342488 | 56 | 3^rd^ | Kaby/Baviacora M92/3/Croc 1/***Ae.Squarrosa*** **(224)**//Opata M85 |
| 169 | 4753159 | BW37868 | 342488 | 57 | 3^rd^ | Kaby/Baviacora M92/3/Croc 1/***Ae.Squarrosa*** **(224)**//Opata M85 |
| 170 | 4753158 | BW37869 | 342488 | 58 | 3^rd^ | Kaby/Baviacora M92/3/Croc 1/***Ae.Squarrosa*** **(224)**//Opata M85 |
| 172 | 4882946 | BW39371 | 363051 | 42 | 3^rd^ | Croc 1/***Ae.Squarrosa*** **(224)**//Opata M85/3/Altar 84/ ***Aegilops squarrosa (Taus)***//Opata M85/4/Pastor*2/Opata M85 |
| 173 | 4883007 | BW39383 | 363192 | 46 | 3^rd^ | Croc 1/***Ae.Squarrosa*** **(224)**//Opata M85/3/Altar 84/ ***Aegilops squarrosa (Taus)***//Opata M85/4/Pastor |
| 174 | 4883006 | BW39384 | 363192 | 49 | 3^rd^ | Croc 1/***Ae.Squarrosa*** **(224)**//Opata M85/3/Altar 84/ ***Aegilops squarrosa (Taus)***//Opata M85/4/Pastor |
| 175 | 4883005 | BW39385 | 363192 | 53 | 3^rd^ | Croc 1/***Ae.Squarrosa*** **(224)**//Opata M85/3/Altar 84/ ***Aegilops squarrosa (Taus)***//Opata M85/4/Pastor |
| 176 | 4885597 | BW39409 | 373340 | 49 | 4^th^ | Ducula//Huitle/Tubeno/3/Carrizo T89/4/Croc 1/***Ae.Squarrosa*** **(224)**//Opata M85/5/Pastor |
| 177 | 4885604 | BW39412 | 373362 | 58 | 3^rd^ | Sterna:Dr/***Ae. Squarrosa*** **(358)**/3/Maioral/Buckbuck//Veery#7/4/Pastor |
| 178 | 4885603 | BW39413 | 373362 | 59 | 3^rd^ | Sterna:Dr/***Ae. Squarrosa*** **(358)**/3/Maioral/Buckbuck//Veery#7/4/Pastor |
| 179 | 4885602 | BW39414 | 373362 | 60 | 3^rd^ | Sterna:Dr/***Ae. Squarrosa*** **(358)**/3/Maioral/Buckbuck//Veery#7/4/Pastor |
| 180 | 4885601 | BW39415 | 373362 | 67 | 3^rd^ | Sterna:Dr/***Ae. Squarrosa*** **(358)**/3/Maioral/Buckbuck//Veery#7/4/Pastor |
| 181 | 4883372 | BW39450 | 365653 | 59 | 5^th^ | Altar 84/***Aegilops Squarrosa*** **(*Taus*)**//Ocoroni F86/3/Veery/Marcos Juarez Inta//2*Tui |
| 182 | 4883371 | BW39451 | 365653 | 60 | 5^th^ | Altar 84/***Aegilops Squarrosa*** **(*Taus*)**//Ocoroni F86/3/Veery/Marcos Juarez Inta//2*Tui |
| 183 | 4883370 | BW39452 | 365653 | 61 | 5^th^ | Altar 84/***Aegilops Squarrosa*** **(*Taus*)**//Ocoroni F86/3/Veery/Marcos Juarez Inta//2*Tui |
| 184 | 4883369 | BW39453 | 365653 | 62 | 5^th^ | Altar 84/***Aegilops Squarrosa*** **(*Taus*)**//Ocoroni F86/3/Veery/Marcos Juarez Inta//2*Tui |
| 185 | 4883367 | BW39454 | 365653 | 64 | 5^th^ | Altar 84/***Aegilops Squarrosa*** **(*Taus*)**//Ocoroni F86/3/Veery/Marcos Juarez Inta//2*Tui |
| 186 | 4878569 | BW39469 | 342452 | 53 | 3^rd^ | Ciano T79//PF70354/Musala/3/Pastor/4/Croc 1/***Ae.Squarrosa*** **(224)**//Opata M85 |
| 187 | 4878716 | BW39477 | 342852 | 77 | 2^nd^ | Sterna:Dr/***Ae. Squarrosa*** **(358)**/4/Ures T81//Buckbuck//Pavon F76/3/Kauz/5/Ures T81/Junco/Kauz |
| 188 | 4878715 | BW39478 | 342852 | 81 | 2^nd^ | Sterna:Dr/***Ae. Squarrosa*** **(358)**/4/Ures T81//Buckbuck//Pavon F76/3/Kauz/5/Ures T81/Junco/Kauz |
| 189 | 4750132 | BW39482 | 335446 | 79 | 3^rd^ | Croc 1/***Ae.Squarrosa*** **(213)**//Papago M86/3/Baviacora M92 |
| 190 | 4750131 | BW39483 | 335446 | 80 | 3^rd^ | Croc 1/***Ae.Squarrosa*** **(213)**//Papago M86/3/Baviacora M92 |
| 191 | 4750130 | BW39484 | 335446 | 81 | 3^rd^ | Croc 1/***Ae.Squarrosa*** **(213)**//Papago M86/3/Baviacora M92 |
| 192 | 4750129 | BW39485 | 335446 | 82 | 3^rd^ | Croc 1/***Ae.Squarrosa*** **(213)**//Papago M86/3/Baviacora M92 |
| 193 | 4750128 | BW39486 | 335446 | 85 | 3^rd^ | Croc 1/***Ae.Squarrosa*** **(213)**//Papago M86/3/Baviacora M92 |
| 196 | 4886016 | BW39489 | 378807 | 61 | 2^nd^ | Decoy 1/***Ae.Squarrosa*** **(458)**/3/Kauz/Gygis/Kauz |
| 197 | 4886015 | BW39490 | 378807 | 63 | 2^nd^ | Decoy 1/***Ae.Squarrosa*** **(458)**/3/Kauz/Gygis/Kauz |
| 198 | 4886014 | BW39491 | 378807 | 67 | 2^nd^ | Decoy 1/***Ae.Squarrosa*** **(458)**/3/Kauz/Gygis/Kauz |
| 199 | 6174895 | BW49397 | 520259 | 21 | 4^th^ | Altar 84/***Ae. Squarrosa*** **(221)**//3*Borlaug M95/3/Ures T81/Junco//Kauz/4/Weebilli/5/Mutus |
| 200 | 6174901 | BW49399 | 520259 | 27 | 4^th^ | Altar 84/***Ae. Squarrosa*** **(221)**//3*Borlaug M95/3/Ures T81/Junco//Kauz/4/Weebilli/5/Mutus |
| 201 |  | BW31795 |  |  |  | Croc 1/***Ae.Squarrosa*** **(662)** |
| 202 |  | BW31800 |  |  |  | Croc 1/***Ae.Squarrosa*** **(662)** |
| 203 |  | BW31813 |  |  |  | Croc 1/***Ae.Squarrosa*** **(879)** |
| 204 |  | BW36899 |  |  |  | Croc 1/***Ae.Squarrosa*** **(224)** |
| **Common wheat** | | **Name of wheat** | |  |  |  |
| 205 |  | Pishtaz | - | - | - |  |
| 206 |  | Roshan | - | - | - |  |
| 207 |  | Kavir | - | - | - |  |
| 208 |  | Ghods | - | - | - |  |
| 1 |  | AAC Scotia | - | - | - |  |
| 2 |  | Carberry | - | - | - |  |
| 4 |  | Hoffman | - | - | - |  |
| 5 |  | Norwell | - | - | - |  |

| **Table S4.** Pedigree of 184 synthetic hexaploid wheats used in this study | | | |
| --- | --- | --- | --- |
| ***T. turgidum* donor** | ***Ae. tauschii* donor** | **No. of lines** | **Primary Synthetic Hexaploid parent** |
| CERCETA | *Ae. searsii* (34D) | 2 | CERCETA/*Ae. searsii* (34D) |
| ALTAR 84 | *Ae. squarrosa* (193) | 1 | ALTAR 84/*Ae. squarrosa* (193) |
| CROC_1 | *Ae. squarrosa* (205) | 9 | CROC_1/ *Ae. squarrosa* (205) |
| Gediz75/3/Goose//Albatros:Dr/Crane | *Ae. squarrosa* (208) | 2 | Gediz75/3/Goose//Albatros:Dr/Crane/*Ae. squarrosa* (208) |
| CROC_1 | *Ae. squarrosa* (213) | 9 | CROC_1/ *Ae. squarrosa* (213) |
| DVERD_2 | *Ae. squarrosa* (214) | 7 | DVERD_2/ *Ae. squarrosa* (214) |
| ALTAR 84 | *Ae. squarrosa* (224) | 1 | ALTAR 84/ *Ae. squarrosa* (224) |
| CROC_1 | *Ae. squarrosa* (224) | 43 | CROC_1/ *Ae. squarrosa* (224) |
| SORA | *Ae. squarrosa* (323) | 5 | SORA/ *Ae. squarrosa* (323) |
| STERNA:DR | *Ae. squarrosa* (358) | 6 | STERNA:DR/ *Ae. squarrosa* (358) |
| YARMUK | *Ae. squarrosa* (434) | 3 | YARMUK/ *Ae. squarrosa* (434) |
| DECOY 1 | *Ae. squarrosa* (458) | 3 | DECOY 1/ *Ae. squarrosa* (458) |
| CROC_1 | *Ae. squarrosa* (662) | 18 | CROC_1/ *Ae. squarrosa* (662) |
| CRANE | *Ae. squarrosa* (895) | 1 | CRANE/ *Ae. squarrosa* (895) |
| ALTAR 84 | *Ae. squarrosa* | 3 | ALTAR 84/ *Ae. Squarrosa* |
| CHEN | *Ae. squarrosa* | 2 | CHEN/ *Ae. Squarrosa* |
| ALTAR 84 | *Ae. squarrosa* (Taus) | 26 | ALTAR 84/ *Ae. squarrosa* (Taus) |
| Cando/R143//Ente/Mexicali_2 | *Ae. squarrosa* (Taus) | 18 | Cando/R143//Ente/Mexicali_2/ *Ae. squarrosa* (Taus) |
| CHEN | *Ae. squarrosa* (Taus) | 10 | CHEN/ *Ae. squarrosa* (Taus) |
| ALTAR84 | *Ae. squarrosa* (219) | 3 | ALTAR84/ *Ae. squarrosa* (219) |
| DECOY 1 | *Ae. squarrosa* (510) | 3 | DECOY 1/ *Ae. squarrosa* (510) |
| CROC_1 | *Ae. squarrosa* (879) | 5 | CROC_1/ *Ae. squarrosa* (879) |
| ALTAR84 | *Ae. squarrosa* (221) | 4 | ALTAR84/ *Ae. squarrosa* (221) |

| **Table S5.** The analysis of field soil related to two growing season of wheat (2018–2019 and 2019–2020) | | | | | | | | | | | | | | | |
| --- | --- | --- | --- | --- | --- | --- | --- | --- | --- | --- | --- | --- | --- | --- | --- |
| **Soil particles (%)** | | | | **Soil texture** | **EC**  **(ds.m^-1^)** | **pH** | **Cu**  **(mg/kg)** | **Mn**  **(mg/kg)** | **Fe**  **(mg/kg)** | **Zn**  **(mg/kg)** | **P**  **(mg/kg)** | **K**  **(mg/kg)** | **N**  **(%)** | **Soil depth**  **(cm)** | **Year** |
| **Clay+Silt** | **Sand** | **Clay** | **Silt** |  |  |  |  |  |  |  |  |  |  |  |  |
| 60 | 40 | 30 | 30 | Clay-Loam | 3.9 | 7.7 | 0.74 | 11.46 | 4.15 | 1.16 | 31.38 | 526.6 | 0.06 | 0-30 | **2018** |
| 63.33 | 36.67 | 33.33 | 30 |  | 2.16 | 7.53 | 0.61 | 6.02 | 4.03 | 0.69 | 19.13 | 483.1 | 0.03 | 30-60 |  |
| - | 41 | 25 | 34 | Loam | 2.1 | 7.70 | 1.3 | 20.1 | 14.9 | 2.5 | 38.6 | 495 | 0.08 | 0-30 | **2019** |
| - | 40 | 26 | 34 | Loam | 1.6 | 7.95 | 0.85 | 7.5 | 10.1 | 0.63 | 6.7 | 300 | 0.03 | 30-60 |  |
| N Nitrogen, K Potassium, P Phosphorus, Zn Zinc, Fe Iron, Mn Manganese, Cu Copper, pH Potential of hydrogen, EC electrical conductivity. | | | | | | | | | | | | | | | |

| b  a  b  a | b |
| --- | --- |
| **a** (DHE) | **b** (RWC) |
| a | a |
| **c** (PHT) | **d** (YLD) |
|  |  |
| **e** (TGW) | **f** (HI) |
| **Figure S1.** Comparison of common and synthetic hexaploid wheats for DHE days to heading (a), RWC (%) relative water content (b), PHT (cm) plant height (c), YLD (g/m^2^) grain yield (d), TGW (g) thousand-grain weight (e), HI (%) harvest index (f).   \|  \| \| --- \| \| **Figure S2.** The trend of temperature and humidity during the growing season of wheat (Oct-Jun) 2018–2019 and 2019–2020. \| | |
